# Development of Novel Therapeutics for Schizophrenia Treatment Based on a Selective Positive Allosteric Modulation of α1-Containing GABAARs—In Silico Approach

**DOI:** 10.3390/cimb44080234

**Published:** 2022-07-29

**Authors:** Vladimir Đorđević, Milan Petković, Jelena Živković, Goran M. Nikolić, Aleksandar M. Veselinović

**Affiliations:** 1 Department of Psychiatry with Medical Psychology, Faculty of Medicine, University of Niš, 18000 Niš, Serbia; vladimir.djordjevic@medfak.ni.ac.rs; 2 Department of Physiology, Faculty of Medicine, University of Niš, 18000 Niš, Serbia; milan.petkovic@medfak.ni.ac.rs; 3 Department of Chemistry, Faculty of Medicine, University of Niš, 18000 Niš, Serbia; jelena.zivkovic.hemija@medfak.ni.ac.rs (J.Ž.); goran.nikolic@medfak.ni.ac.rs (G.M.N.)

**Keywords:** GABAA receptor, schizophrenia, QSAR, molecular modeling, drug design

## Abstract

For the development of atypical antipsychotics, the selective positive allosteric modulation of the ionotropic GABAA receptor (GABAAR) has emerged as a promising approach. In the presented research, two unrelated methods were used for the development of QSAR models for selective positive allosteric modulation of 1-containing GABAARs with derivatives of imidazo [1,2-a]-pyridine. The development of conformation-independent QSAR models, based on descriptors derived from local molecular graph invariants and SMILES notation, was achieved with the Monte Carlo optimization method. From the vast pool of 0D, 1D, and 2D molecule descriptors, the GA-MLR method developed additional QSAR models. Various statistical methods were utilised for the determination of the developed models’ robustness, predictability, and overall quality, and according to the obtained results, all QSAR models are considered good. The molecular fragments that have a positive or negative impact on the studied activity were obtained from the studied molecules’ SMILES notations, and according to the obtained results, nine novel compounds were designed. The binding affinities to GABAAR of designed compounds were assessed with the application of molecular docking studies and the obtained results showed a high correlation with results obtained from QSAR modeling. To assess all designed molecules’ “drug-likeness”, their physicochemical descriptors were computed and utilised for the prediction of medicinal chemistry friendliness, pharmacokinetic properties, ADME parameters, and druglike nature.

## 1. Introduction

Of all the psychiatric illnesses, schizophrenia is recognised as one of the most serious. This is a complex, chronic mental disorder affecting approximately 1% of the world population. Illness symptoms range from delusions to hallucinations to disorganised thinking and impaired cognitive ability [1,2]. The disorder is modestly more common in men than in women [3]. For many patients and their families, the early onset of schizophrenia and its chronic nature make schizophrenia a very disabling disorder [4,5]. The life expectancy of schizophrenia patients is reduced by approximately ten years, mainly by suicide. Even if the recovery prognosis is considered more optimistic today, only a minority of patients are still fully recovered [6]. Among those who have good outcomes, the diagnosis has life−changing consequences, including social isolation and stigma [3].

Antipsychotic agents are necessary for the effective rehabilitation of most schizophrenia patients. Antipsychotics are the mainstay of pharmacological therapy, but they are associated with side effects that can cause disability [1]. Gamma-aminobutyric acid (GABA) is the most abundant inhibitory neurotransmitter in the mammalian central nervous system, and its distribution suggests that it plays a significant role in virtually all brain physiological functions, as well as transmission in neurodevelopmental disorders [7]. The GABAA receptor is one of the principal drug targets in the treatment of neuropsychiatric disorders such as insomnia, anxiety, epilepsy, and anaesthesia in surgical interventions [8]. This type of GABA receptor serves as the target of numerous classes of drugs [7]. There is strong evidence implicating impairments of the GABA signalling mechanism in the pathophysiology of schizophrenia [9]. Postmortem studies have provided confirmation of an altered GABA system in its pathophysiology [10].

In the drug discovery and development process, research time and financial aspects have a high impact. Both of these limiting factors could be highly influenced by the application of in silico methods since these methods accelerate research and reduce financing by discarding unfavourable molecules. For instance, quantitative structure-activity relationship (QSAR) studies are aimed at developing a mathematical model that can predict the studied activity for molecules that have not been synthesised and discarding molecules with undesired activity. Not only can in silico methods be used to predict studied activity, but also they could be used to predict and optimise pharmacological activity (or pharmacokinetic properties), key parameters for clinical approval [11,12]. The QSAR modelling process can be summarised as the development of a mathematical correlation, in most cases represented as a mathematical equation, between molecular descriptors and studied activity, where molecular descriptors are numerical parameters of each molecule calculated from its defined molecular structure. It has to be noted that currently a vast number of methodologies are used for QSAR modelling purposes, with their strengths and weaknesses derived from different molecular descriptors and with the application of different computational algorithms used for model development [13,14,15].

The main aim of the presented research was the development of QSAR models for positive allosteric modulation of the ionotropic GABAA receptor. Two approaches were used for this purpose: first based on the Monte Carlo optimization method and conformation−independent molecular descriptors (both SMILES notation and local graph invariants were used), and second based on a genetic algorithm coupled with multiple linear regression and 0D, 1D, and 2D molecular descriptors, with further determination of molecular fragments or structural requirements responsible for allosteric modulation of the ionotropic GABAA receptor. For the “final validation” of the developed QSAR models and designed molecules’ allosteric modulation potential, molecular docking studies were used and the binding affinities to GABAAR of designed compounds were assessed.

## 2. Material and Methods

### 2.1. Molecules Database

**Figure 1:**
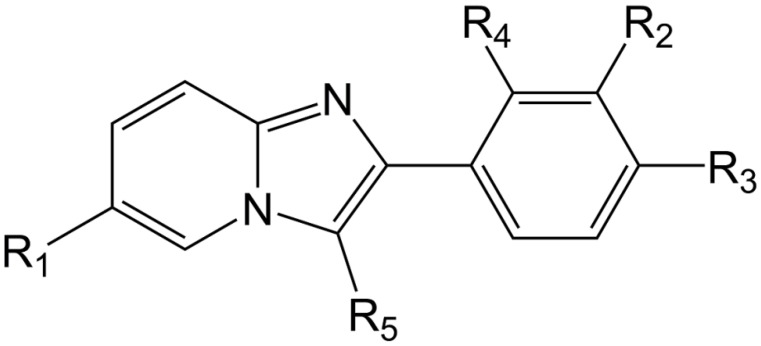
General chemical structure of studied molecules.

The literature provided a data set of 33 imidazo [1,2-a]-pyridine derivatives as selective positive allosteric modulators of α1-containing GABAARs [16]. The anti-psychotic activities, related to positive allosteric modulation of α1-containing GABAARs and expressed in nM, were converted into the corresponding pKi (pKi = −log10 Ki) values, which are used as dependent variables in this study. The chemical structures of all used molecules are represented with appropriate SMILES notation with their pKi listed in Table S1 (see Supplementary Materials), while their general chemical structure is presented in Figure 1. One of the main requirements for developing QSAR models with the Monte Carlo optimization method is that SMILES notation for all molecules be canonized in the same manner. For this purpose, all molecules were drowned using ACD/ChemSketch 2018 v2.5 (Toronto, ON, Canada). The same software was used to obtain the SMILES notation of all molecules. Final SMILES notation canonization was performed using OpenBabel v2.4.1 (Pittsburgh, PA, USA). From the original data set, three random splits into the training (25 compounds, 75%) and test set (8 compounds, 25%) were generated, and for all splits, the normality of the activity distribution was checked according to the literature [17].

### 2.2. The Monte Carlo Optimization Method

Molecular descriptors derivated from both SMILES notation and molecular graph were used to develop conformation-independent QSAR models with the Monte Carlo optimization method as the main computer algorithm. Local graph invariants with the most elementary concepts like walks and paths were used as molecular graph-based descriptors and their detailed mathematical definition can be found in the literature [18]. As optimal topological descriptors, the following local graph invariants were used in the presented research: the number of carbon atom neighbors (Number of Carbon) and the number of non-carbon atom neighbors (Number of Non Carbon), Morgan extended connectivity indices (EC0), valence shells of range 2 and 3 (s2, s3), and path numbers of length 2 and 3 (p2, p3). One of the main downfalls of molecular topological descriptors is the lack of mechanistic interpretation. To overcome this issue, SMILES notation based molecular descriptors are used since correlation with molecular fragments can be made. In the Monte Carlo optimization method, each descriptor, both graph-based and SMILES notation based, is assigned an appropriate numerical value defined as correlation weights (CW). For each molecule, its correlation weight (DCW) is the sum of each descriptor’s CW and its calculation is presented in Equation (1).

DCW(T,N_epoch_) = ΣCW(S_k_) + ΣCW(SS_k_) + ΣCW(SSS_k_) + ΣCW(ATOMPAIR) + ΣCW(NOSP) + ΣCW(BOND) + ΣCW(HALO) + ΣCW(PT2_k_) + ΣCW(PT3_k_) + ΣCW(VS2_k_) + ΣCW(VS3_k_) + ΣCW(NNC_k_) (1)

A SMILES atom defined as one SMILES notation symbol (or two un-separated symbols) is represented as S_k_, while symbols SS_k_ and SSS_k_ represent the linear combinations of two and three SMILES atoms, respectively. The global features of the studied molecule, defined with global SMILES notation, used in this research were: HARD, NOSP, BOND, HALO, and ATOMPAIR, all calculated according to the published methodology [19,20]. In this research, a hybrid approach was used where the combination of both local graph invariant descriptors and SMILES notation (both local and global) was used for QSAR model development.

Apart from the above defined S_k_, SS_k_ and SSS_k_ symbols in Equation (1), the following descriptors were represented with appropriate symbols: EC0_k_—Morgan connectivity index of zero order with hydrogen suppressed graph; VS2_k_ and VS3_k_—valence shell 2 and 3; PT2_k_ and PT3_k_—paths of length 2 and 3; NNC_k_—Nearest Neighbors [18]. CORAL software (CORrelation and Logic) (Milano, Italy). (http://www.insilico.eu/coral (accessed on 12 February 2022)) was used for QSAR model development based on the Monte Carlo optimization method and for the calculation of all above−defined molecular descriptors. Publish methodology [19,20] was used for QSAR development, and a detailed description of the model development process is given in the Supplementary Materials.

### 2.3. GA-MLR Method

PaDEL software was used for the calculation of molecular descriptors used for QSAR modeling based on genetic algorithms coupled with multiple linear regression (GA-MLR) [21]. Descriptors with low variance were discarded from the initial descriptor pool. Further descriptors number reduction was achieved by filtering based on high pairwise intercorrelation coefficients-descriptors (descriptors with 80% constant value and 95% correlation were omitted from QSAR model development). After the reduction of descriptor number and their scaling, QSAR models were developed with the application of genetic algorithm (GA) optimization method coupled with multiple linear regression (MLR). The QSAR model was established for the split into training and test sets that produced the best statistical parameters within the Monte Carlo optimization method. For the QSAR model development based on GA-MLR approach, the QSARINS program (QSAR-INSUBRIA) (www.qsar.it (accessed on 5 March 2022)) was used. This software was also used for the reduction of descriptor numbers [22,23]. The following parameters GA-MLR were set in the QSAR model development: the total number of features in the model (GA optimization included the number of variables) was 5, the number of GA iterations (generations per size) was 500, the number of models on which GA evolves (population size) was 10, and random mutations to generate a pool of variegated descriptors (mutation rate) were 20%.

### 2.4. QSAR Models Validation and Applicability Domain

The goodness of developed QSAR models, both with the Monte Carlo optimization and GA-MLR method, was assessed with the application of several validation metrics: squared correlation coefficient (r^2^); leave-one-out and leave-many-out cross-validation coefficients ($q^{2_{loo}}$, q^2^); root-mean-squared-error (RMSE), mean absolute error (MAE), F-value, and y-scrambling. In addition to above stated parameters, correlation coefficient (CCC), the Index of Ideality of Correlation (IIC), MAE-based metrics and R_m_^2^ were used for further developed QSAR models validation [19,20,24,25,26,27,28,29].

Applicability domain (AD) is considered as a crucial addition to any reliable, relevant, valid and robust QSAR model, and it is a feature that must be defined before the QSAR model is developed fully [30,31]. For QSAR models developed with the Monte Carlo optimization method, the approach with the “statistical defects” of conformation-independent molecular descriptors–*d*(A) was used for the determination of AD [17]. Williams plot (standardised residuals versus leverages), a distance−based method, was used for defining AD of models developed with GA-MLR.

### 2.5. Molecular Docking

As the target for docking studies, human 122 GABAAR (PDB: 6X3X) was selected and Molegro Virtual Docker (MVD) (Odder, Denmark) was used as the main software. MVD provides information for both hydrophilic interactions, including the identification of hydrogen bonds between rigid amino acids from defined active sites and studied ligands, and hydrophobic (mostly related to steric and Van der Waals interactions), by employing docking studies between flexible ligands and rigid amino acids within the studied enzyme’s active site. Calculated numerical values related to relevant binding energies, defined as “scoring” functions, can be used to quantify the above-stated interactions [32].

The potential inhibition effect of studied ligands could be used to assess obtained numerical values for “scoring” functions, since, for most enzymes, the higher the interaction between ligand and receptor, the higher is the inhibition observed [20]. The following “scoring” functions were calculated and used for inhibitory potential estimation: MolDock, and Rerank Score, Pose energy, VdW, Steric, NoHbond, and Hbond. Published methodology was used to validate a complete molecular docking protocol [33,34]. Discovery Studio Client v20.1.0.19. (Waltham, MA, USA) was used for two−dimensional representations of the interactions between the amino acids from α1-containing the GABAARs active site and studied molecules.

## 3. Results and Discussion

### 3.1. The Monte Carlo Optimization Method

**Figure 2:**
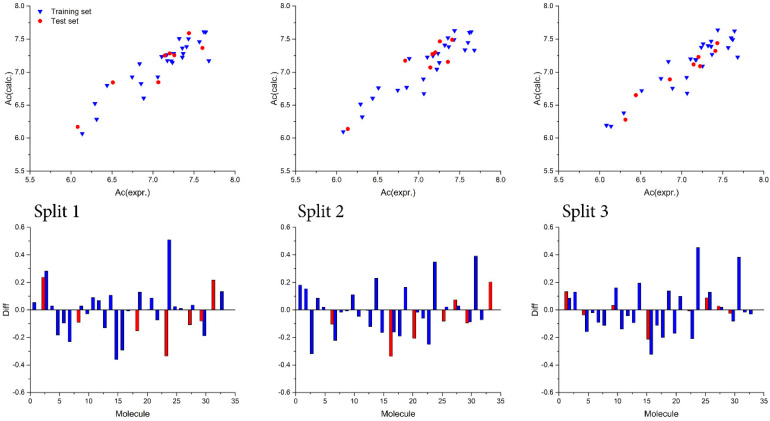
(**Above**): graphical representation of the best Monte Carlo optimization runs (the highest value for the r^2^) for the developed QSAR models; (**Below**): difference between experimental and calculated values for pKi.

The assessment of developed QSAR conformation−independent models’ robustness, predictability, and overall quality several statistical metrics were calculated and their numerical values are presented in Table 1, including: r^2^—correlation coefficient, q^2^—cross-validated correlation coefficient, s—standard error of estimation, MAE—Mean absolute error and F—Fischer ratio. Table 1 presents numerical values for CCC—concordance correlation coefficient are IIC—index of ideality of correlation. Numerical values for all statistical matrices indicate that QSAR models obtained with the application of the Monte Carlo optimization method have good reproducibility and high predictability potential. Applied methodology for AD suggested that no outliers were present, since all molecules were within the defined AD and all were used for both model development and model testing. The highest obtained r^2^ value was used to assess the best developed QSAR model, regarding the best Monte Carlo optimization run, and a graphical representation of these QSAR models for all three splits, is presented in Figure 2. Further, the difference between calculated values and experimental values for studied activity, for both molecules in the training and test set, is presented in Figure 2. For further validation of developed QSAR models, especially determination of their reproducibility, concordance correlation coefficients (CCC) were calculated and according to the obtained numerical values for CCC suggest that all developed QSAR models possess high reproducibility. The developed QSAR models were finally validated with the calculation of an MAE—based metric and the obtained results indicated models as GOOD. Y—randomization was used to determine developed QSAR models’ sturdiness, and according to the obtained results, presented in Table S2 (Supplementary Material), calculated QSAR models are free from correlation by chance. Finally, the predictive potential of calculated QSAR models was assessed with the index of ideality of correlation (IIC); high numerical values calculated for IIC indicate that QSAR models developed with the Monte Carlo optimization method have high predictive potential.

Mathematical representation of the best QSAR models developed with Monte Carlo optimization according to obtained test set r^2^ for all splits are given in Equations (2)–(4).

Split 1: pK_i_ = −1.0585(±0.1320) + 0.0612(±0.0010) × DCW(2,11) (2)

Split 2: pK_i_ = 0.7626(±0.0977) + 0.0414(±0.0006) × DCW(3,12) (3)

Split 3: pK_i_ = −3.2411(±0.1623) + 0.0948(±0.0015) × DCW(3,7) (4)

According to Equations (2)–(4), preferable values for T and N_epoch_ are 2 and 11 for split 1, respectively; preferable values for T and N_epoch_ for split 2 are 3 and 12, respectively; and preferable values for T and N_epoch_ for split 3 are 3 and 7, respectively.

### 3.2. GA-MLR Modeling

Equation (5) presents mathematical equations for the QSAR model obtained from GA−MLR modeling and in Supplementary Material its graphical representation is given (Figure S1). The Supplementary Material (Table S3) also presents numerical values for metrics used for the developed QSAR model validation, and according to the obtained results the developed QSAR model has satisfactory prediction potential and its prediction can be considered as robust.

pK_i_ = 6.4712 − 0.2443 × ATSC4p − 0.0345 × AATSC8m − 5.1954 × SpMin7_Bhp + 7.0601 × SpMin8_Bhi + 1.2733 × topoShape (5)

According to presented Equation (5), molecular descriptors that have importance for model development are the following: ATSC4p—centered Broto−Moreau autocorrelation—lag 4/weighted by polarizabilities; AATSC8c—Average centered Broto−Moreau autocorrelation—lag 8/weighted by charges; SpMin7_Bhp—Smallest absolute eigenvalue of Burden modified matrix—n 7/weighted by relative polarizabilities; SpMin8_Bhi—Smallest absolute eigenvalue of Burden modified matrix—n 8/weighted by relative first ionization potential and topoShapePetitjean topological shape index.

### 3.3. Comparison to the Other QSAR Models

Zheng et al. (2021) investigated novel selective positive allosteric modulators (PAMs) of 1-containing GABAARs with an imidazo [1,2-a]-pyridine heterocyclic system [16]. The research authors developed three−dimensional quantitative structure-activity relationships (3D-QSAR) and performed pharmacophore modelling as well as molecular docking and molecular dynamics studies for 33 imidazo [1,2-a]-pyridines in the search for novel antipsychotic drugs and to obtain better understanding of their pharmacological characteristics. The constructed 3D-QSAR models showed good predictive potential, and the obtained results could provide a significant basis for new potent antipsychotics. In comparison to the QSAR models presented in research performed by Zheng et al., the presented QSAR models have similar predictability according to comparison of numerical values of the used statistical metrices. However, QSAR models presented in this research have some advantages mostly related to calculation time and computational resources, since development of 3D QSAR models is both time-consuming and requires advanced computational resources. One of the key steps in the development of 3D based QSAR models is obtaining an appropriate molecule’s geometry and further alignment; both of these steps are skipped in conformational-independent QSAR modelling. Further Monte Carlo optimization methods can be used for the defining of SMILES notation optimal descriptors that could be associated with appropriate molecular fragments, with effect on the studied activity, and this cannot be done with 3D QSAR modelling [19,20].

### 3.4. Computer-Aided Design of Novel Inhibitors

The calculation example of a molecule’s summarized correlation weight (DCW) related to appropriate molecular fragment contribution on pKi is presented in Table 2. In Table 2 molecular graph-based descriptors were omitted to achieve an easier interpretation, while the full list of molecular descriptors, both based on the molecular graph and the SMILES notation is shown in Table S4 (Supplementary Material).

**Figure 3:**
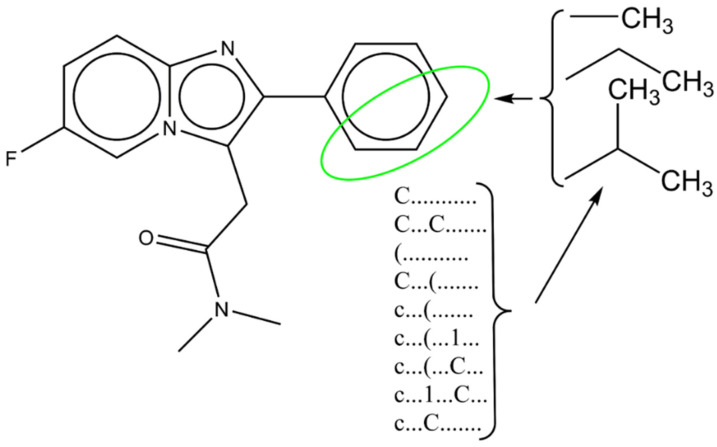
Schematics for computer—aided drug design.

In the process of computer−aided design (CAD), the results from conformational−independent studies, mostly related to defined molecular fragments, were used for the design of nine novel potential inhibitors, whose structures are presented in Figure 3. Figure 3 also presents simplified schematics related to CAD.

As a template molecule (A), one of the least chemically exploited molecules from the original dataset (molecule 3) was chosen. After examination of the molecules dataset, the observation was made that the phenyl group is the least chemically modified, so CAD was focused on the addition of simple aliphatic groups in ortho, meta, and para positions. Only molecular fragments with a positive impact on pKi were selected. Results for CAD molecules with calculated pKi are presented in Table 3.

In the three positions, molecular fragments that have a positive impact on pKi have been added and lead to an increase in pKi: “C..........”—a methyl group or simple carbon atom; “c...(...1...”; “c...(...C...”; These SMILES notation descriptors are associated with a molecule’s branching resulting from the addition of at least one methyl group to benzene. All designed molecules had a higher pKi in comparison to the molecule’s average pKi. Between molecules A1–A9, a difference in calculated pKi can be observed, where the highest pKi was obtained for molecule A7 while the lowest was obtained for molecule A2. The possible explanation for this observation can be associated with the number of molecular fragments−SMILES notation descriptors. Molecule A7 has the highest number of molecular fragments with a positive impact. Molecular fragment “c...” has a negative impact on pKi, reducing its numerical value. Molecule A2 has that fragment, while it is absent in molecule A7. If calculated values for molecule A7, A8 and A9 are compared, both A8 and A9 have lower values and both have “c...” (..c...” fragment.

### 3.5. Molecular Docking

**Figure 4:**
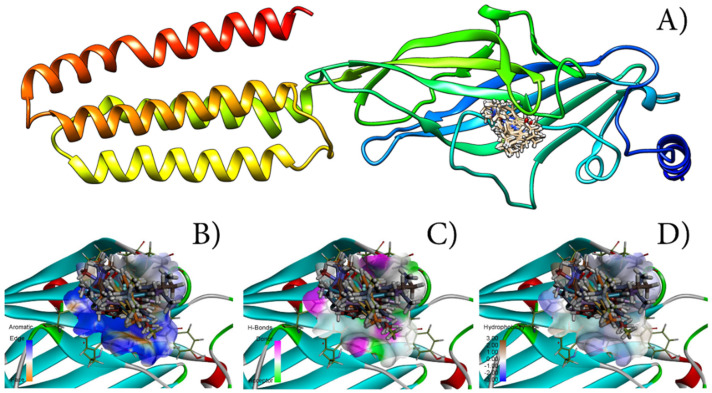
(**A**) Best calculated poses for all designed molecules inside the active site of α1-containing GABAARs; (**B**) Aromatic interactions inside active site; (**C**) Hydrogen bonds related interactions inside active site; (**D**) Hydrophobic interactions inside active site.

One of the hypotheses given in this research is that the stronger the binding of ligand with amino-acid from the receptor’s active site is (quantified as binding energy, which can be correlated with “score” functions), the more active the ligand is (quantified as pKi). To assess this hypothesis and to evaluate developed QSAR models’ predictability and validate them further, molecule A and all designed molecules were subjected to molecular docking studies with α1-containing GABAAR. It has to be noted that the docking protocol used in this research was validated with an appropriate protocol. This protocol was RMSD based and for all molecules, where RMSD values for all studied molecules were compared to co−crystallized ligand (diazepam) and the obtained results were lower than 1.5, validating all docking poses. In Table 4, numerical values for all calculated “scoring” functions are presented. To assess the possible inhibitory potency of a ligand, different physical−chemical interactions between the ligand and amino acids should be taken into consideration. The potential for the highest inhibitory activity can be associated with the highest MolDock and ReRank “score” functions values, calculated for molecule A7. This observation is in good correlation with the results obtained from QSAR modelling and CAD, where the highest pKi was calculated for molecule A7 also. Further, the highest energy related to hydrogen bond interaction was observed for molecule A7, while the lowest was observed for molecule A8. The impact of other “scoring” functions on potential inhibitory activity could be determined in similar manner as for MolDock and ReRank “score” functions according to [20]. Performed molecular docking studies identified hydrophobic, hydrophilic interactions as well as hydrogen bonds, between designed molecules and amino acids inside the binding pocket of α1-containing GABAAR, and they are presented in Figure 4 as the most preferable geometric orientation (molecular pose) inside the active site. 2D representations of these interactions are presented in the Figures S2–S11 in the Supplementary Materials. Molecular docking studies identified the following amino acids as important: TYR160—all molecules interacted with it via-alkil and/or -stacking interactions; TYR210 and SER205 formed hydrogen bonds.

### 3.6. ADME Determination

**Figure 5:**
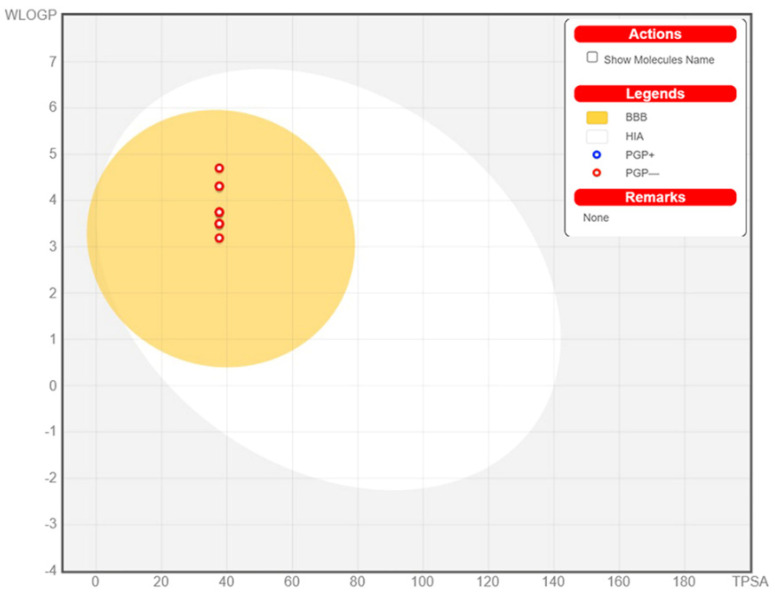
BOILED−Egg diagram for all designed molecules (BBB—Blood–brain barrier; PG+/−—P−glycoprotein; HIA—human intestinal absorption).

The determination of physicochemical characteristics of studied molecules is one of the first steps in the early stages of drug development preferably done with the application of computational methods and for the purpose of determining whether new compounds have features that will classify them as potential therapeutics. To classify a molecule as drug-like, it has to possess the efficacy of binding to receptors/channels, oral bioavailability, gastrointestinal absorption, optimal bioavailability, good absorption/permeation, and brain penetration. All of these features could be predicted by knowing the molecule structure, so the molecule can be discarded from further development before it is synthesized. The computation of physicochemical descriptors as well as the prediction of medicinal chemistry friendliness, druglike nature, pharmacokinetic properties, and ADME parameters of designed molecules were computed with the SwissADME web service for the purpose of drug-likeness evaluation [35], and the obtained results are presented in Table S5 (Supplementary Material). All designed molecules possess high drug-likeness according to obtained results, which can also be observed in graphical representations of designed molecules’ important physicochemical features named as Bioavailability Radars, that are presented in the Supplementary Material (Figures S12–S21). Gastro-interstitial absorption and brain access are two pharmacokinetic behaviors crucial to estimating favorable profiles for studied molecules. In this research, a predictive accurate model based on computing the lipophilicity and polarity of small molecules named the Brain Or IntestinaL EstimateD permeation method (BOILED-Egg) [36] is applied and the obtained results are presented in Figure 5. As presented in Figure 5 all designed molecules have favorable gastrointestinal absorption and brain access profiles since they are located in the egg yolk, part of BOILED-Egg.

## 4. Conclusions

The main aim of this research was to develop robust QSAR models with good predictability, determined using numerous statistical parameters, for a selective positive allosteric modulation of 1-containing GABAARs. The Monte Carlo optimization method was used to calculate conformation-independent models, developed based on optimal descriptors derived both from a local graph and the SMILES notation invariants. A genetic algorithm coupled with multiple linear regression was used to obtain a QSAR model from the pool of vast 0D, 1D, and 2D molecule descriptors. The evaluation of the developed QSAR models’ robustness and predictive potential was executed by applying a range of statistical parameters, and their numerical values indicate that all developed QSAR models possess high predictability. The SMILES notation descriptors, defined as molecular fragments, with their influence on selective positive allosteric modulation of 1-containing GABAARs, were defined with the application of the Monte Carlo optimization method and are used for computer-aided design of novel compounds that have higher pKi values in comparison to starting template molecules. The binding preferences of designed molecules with amino acids from the active sites of 1-containing GABAARs were determined with the application of molecular docking studies. Molecular docking studies were used to calculate “scoring functions” related to binding energies that were used further to assess the effects on 1-containing GABAARs in regards to potential selective positive allosteric modulation. According to the obtained results, good inter-correlation can be observed between molecular docking studies and developed QSAR models regarding their potential selective positive allosteric modulation. Designed molecules’ physicochemical descriptors were computed to predict medicinal chemistry friendliness, pharmacokinetic properties, druglike nature, and ADME parameters, and according to the obtained results, all designed molecules possess high drug-likeness. Further, high bioavailability, including gastrointestinal absorption and brain access, of designed molecules was determined. Results presented in this research could be used in the search for novel antipsychotic agents for schizophrenia treatment whose main target activity is selective positive allosteric modulation of 1-containing GABAARs.

## Figures and Tables

**Table 1:** The statistical quality of QSAR models developed with the Monte Carlo optimization method for selective positive allosteric modulation of α1-containing GABAARs.

Run		Training Set							Test Set						
		r^2^	CCC	IIC	q^2^	s	MAE	F	r^2^	CCC	IIC	q^2^	s	MAE	F
Split 1	1	0.8553	0.922	0.7267	0.8288	0.166	0.116	136	0.8468	0.9192	0.9202	0.7767	0.207	0.163	33
	2	0.8302	0.9073	0.7159	0.8059	0.180	0.127	112	0.8586	0.9164	0.9266	0.7872	0.194	0.152	36
	3	0.8413	0.9138	0.8467	0.8104	0.174	0.118	122	0.8355	0.9141	0.9140	0.7468	0.209	0.163	30
	Av	0.8423	0.9144	0.7631	0.8150	0.173	0.120	123	0.8470	0.9166	0.9203	0.7702	0.203	0.159	33
Split 2	1	0.8518	0.9200	0.7252	0.8296	0.179	0.139	132	0.8523	0.9086	0.9225	0.6671	0.181	0.138	35
	2	0.8219	0.9023	0.8369	0.7888	0.196	0.159	106	0.8419	0.9123	0.9175	0.6644	0.164	0.114	32
	3	0.8483	0.9179	0.6140	0.8223	0.181	0.147	129	0.8371	0.9118	0.9145	0.6309	0.171	0.117	31
	Av	0.8407	0.9134	0.7254	0.8136	0.185	0.148	122	0.8438	0.9109	0.9182	0.6541	0.172	0.123	33
Split 3	1	0.8411	0.9137	0.6114	0.8178	0.182	0.143	122	0.9479	0.9678	0.9735	0.8732	0.103	0.070	109
	2	0.8528	0.9205	0.6157	0.8283	0.175	0.132	133	0.9164	0.9456	0.9573	0.7794	0.145	0.121	66
	3	0.8557	0.9223	0.8539	0.8365	0.174	0.140	136	0.9121	0.9530	0.9540	0.8476	0.131	0.104	62
	Av	0.8499	0.9880	0.6937	0.8275	0.177	0.138	130	0.9255	9.5555	0.9616	0.8334	0.126	0.098	79

r^2^—Correlation coefficient; CCC—concordance correlation coefficient; IIC—index of ideality of correlation; q^2^—Cross−validated correlation coefficient; s—Standard error of estimation; MAE—Mean absolute error; F—Fischer ratio; Av—Average value for statistical parameters obtained from three independent Monte Carlo optimization runs.

**Table 2:** The example of DCW(3,7) calculation.

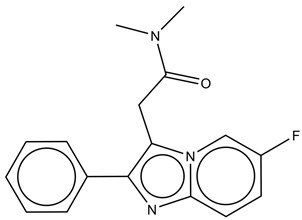 SMILES notation: Fc1ccc2n(c1)c(CC(=O)N(C)C)c(n2)c1ccccc1 DCW = 105.42778 Prediction for EndPoint = 6.7559							
**SA**	**CW(SA)**	**SA**	**CW(SA)**	**SA**	**CW(SA)**	**SA**	**CW(SA)**
F...........	0.0034	1...........	0.0539	c...(.......	0.4537	(...N...(...	0.0812
c...........	0.0736	c...........	0.0736	c...(.......	0.4537	N...(...C...	1.0483
1...........	0.0539	c...........	0.0736	n...(.......	0.5428	(...C...(...	−0.5696
c...........	0.0736	c...........	0.0736	n...2.......	0.2752	C...(...C...	0.8183
c...........	0.0736	c...........	0.0736	2...(.......	0.8207	(...C...(...	−0.5696
c...........	0.0736	c...........	0.0736	c...(.......	0.4537	c...(...C...	−0.4617
2...........	4.4302	1...........	0.0539	c...1.......	0.0753	(...c...(...	2.227
n...........	1.3439	c...F.......	0.2931	c...1.......	0.0753	n...(...c...	0.3335
(...........	−0.9688	c...1.......	0.0753	c...c.......	0.3591	2...n...(...	−0.7845
c...........	0.0736	c...1.......	0.0753	c...c.......	0.3591	n...2...(...	0.0209
1...........	0.0539	c...c.......	0.3591	c...c.......	0.3591	c...(...2...	0.3706
(...........	−0.9688	c...c.......	0.3591	c...c.......	0.3591	1...c...(...	1.2055
c...........	0.0736	c...2.......	−0.5812	c...1.......	0.0753	c...1...c...	0.4915
(...........	−0.9688	n...2.......	0.2752	F...c...1...	−0.2005	c...c...1...	1.4286
C...........	−0.116	n...(.......	0.5428	c...1...c...	0.4915	c...c...c...	0.7319
C...........	−0.116	c...(.......	0.4537	c...c...1...	1.4286	c...c...c...	0.7319
(...........	−0.9688	c...1.......	0.0753	c...c...c...	0.7319	c...c...c...	0.7319
=...........	1.1592	1...(.......	0.3914	c...c...2...	1.1064	c...c...1...	1.4286
O...........	−0.0923	c...(.......	0.4537	n...2...c...	0.3482	Cmax.2......	0.8467
(...........	−0.9688	c...(.......	0.4537	2...n...(...	−0.7845	Nmax.1......	0.0445
N...........	−0.0466	C...(.......	0.2144	n...(...c...	0.3335	Omax.1......	0.0472
(...........	−0.9688	C...C.......	0.0285	1...c...(...	1.2055	Smax.0......	2.741
C...........	−0.116	C...(.......	0.2144	c...1...(...	0.0569	NOSP11000000	0.0558
(...........	−0.9688	=...(.......	2.1975	c...(...1...	−0.1945	HALO10000000	0.5334
C...........	−0.116	O... = .......	1.9829	(...c...(...	2.227	BOND10000000	−0.2316
(...........	−0.9688	O...(.......	2.1172	c...(...C...	−0.4617	++++F−−−N===	0.6827
c...........	0.0736	N...(.......	0.0575	C...C...(...	0.0563	++++F−−−O===	0.7066
(...........	−0.9688	N...(.......	0.0575	C...C...(...	0.0563	++++N−−−O===	1.2605
n...........	1.3439	C...(.......	0.2144	C...(...=...	1.221	++++F−−−B2==	−0.952
2...........	4.4302	C...(.......	0.2144	O...=...(...	0.6219	++++O−−−B2==	0.2153
(...........	−0.9688	C...(.......	0.2144	=...O...(...	2.0622	++++N−−−B2==	1.4959
c...........	0.0736	C...(.......	0.2144	O...(...N...	−0.4948	10011001000	0.3564

**Table 3:** The list of all designed molecules with their SMILES notation and calculated activities.

Molecule	SMILES Notation	pKi
A	Fc1ccc2n(c1)c(CC(=O)N(C)C)c(n2)c1ccccc1	6.7559
A1	Fc1ccc2n(c1)c(CC(=O)N(C)C)c(n2)c1ccccc1C	7.4613
A2	Fc1ccc2n(c1)c(CC(=O)N(C)C)c(n2)c1cccc(c1)C	7.1357
A3	Cc1ccc(cc1)c1nc2n(c1CC(=O)N(C)C)cc(cc2)F	7.5160
A4	CCc1ccccc1c1nc2n(c1CC(=O)N(C)C)cc(cc2)F	7.1291
A5	CCc1cccc(c1)c1nc2n(c1CC(=O)N(C)C)cc(cc2)F	7.3736
A6	CCc1ccc(cc1)c1nc2n(c1CC(=O)N(C)C)cc(cc2)F	7.3465
A7	CCN(C(=O)Cc1c(nc2n1cc(F)cc2)c1ccccc1C(C)C)C	8.0238
A8	Fc1ccc2n(c1)c(CC(=O)N(C)C)c(n2)c1cccc(c1)C(C)C	7.7217
A9	Fc1ccc2n(c1)c(CC(=O)N(C)C)c(n2)c1ccc(cc1)C(C)C	7.6353

**Table 4:** Score values (kcal/mol) for all computer−aided designed compounds.

Molecule	Steric	VdW	HBond	NoHBond90	Energy	MolDock Score	Rerank Score
A	−140.362	−46.2960	−6.23697	−6.65395	−141.363	−139.441	−117.842
A1	−145.212	−12.2465	−3.76233	−4.03557	−145.993	−137.563	−86.1434
A2	−145.305	−44.9493	−6.96824	−7.94622	−148.877	−146.146	−121.477
A3	−140.519	−42.7464	−6.89116	−7.66588	−143.953	−140.793	−117.046
A4	−151.161	−42.7453	−2.49702	−2.50000	−145.106	−143.846	−115.584
A5	−151.189	−46.0394	−3.74132	−4.28093	−152.537	−151.130	−120.893
A6	−150.538	−21.8530	−4.48654	−6.94755	−146.135	−146.097	−108.561
A7	−148.919	−26.6788	−7.14605	−7.95491	−152.918	−153.347	−117.813
A8	−157.365	−39.4826	−2.50000	−2.50000	−154.518	−150.102	−110.772
A9	−151.100	−17.1298	−4.53151	−7.03151	−146.090	−148.784	−105.663

## Data Availability

Data is contained within the article and Supplementary Materials.

## Supplementary Materials

The following supporting information can be downloaded at: https://www.mdpi.com/article/10.3390/cimb44080234/s1. Table S1: The SMILES notation of the studied molecules, calculated values for the DCW, experimental data (Ac)—expr, the values of Ac calculated with the application of CORAL software—calc, the difference between expr and calc—diff for the built QSPR model; Table S2. Y-randomization of the best QSAR model (best optimization run) for three independent splits; Table S3. The statistical quality of QSAR models developed with the GA-MLR method for selective positive allosteric modulation of α1-containing GABA_A_Rs; Table S4. The list of SAks together with their correlation weights for the three runs of the Monte Carlo optimization; Table S5. Calculated physical-chemical parameters for drug-likeness evaluation; Figure S1. (Above left) Graphical representation of developed QSAR model with GA-MLR method for split 3; (Above right) Difference between experimental and calculated pKi values; (Bellow) Graphical representation of applicability domain established for split 3; Figure S2. Two-dimensional representation of the interaction between molecule A and amino acids inside ionotropic GABA_A_ receptor; Figure S3. Two-dimensional representation of the interaction between molecule A1 and amino acids inside ionotropic GABA_A_ receptor; Figure S4. Two-dimensional representation of the interaction between molecule A2 and amino acids inside ionotropic GABA_A_ receptor; Figure S5. Two-dimensional representation of the interaction between molecule A3 and amino acids inside ionotropic GABA_A_ receptor; Figure S6. Two-dimensional representation of the interaction between molecule A4 and amino acids inside ionotropic GABA_A_ receptor; Figure S7. Two-dimensional representation of the interaction between molecule A5 and amino acids inside ionotropic GABA_A_ receptor; Figure S8. Two-dimensional representation of the interaction between molecule A6 and amino acids inside ionotropic GABA_A_ receptor; Figure S9. Two-dimensional representation of the interaction between molecule A7 and amino acids inside ionotropic GABA_A_ receptor; Figure S10. Two-dimensional representation of the interaction between molecule A8 and amino acids inside ionotropic GABA_A_ receptor; Figure S11. Two-dimensional representation of the interaction between molecule A9 and amino acids inside ionotropic GABA_A_ receptor; Figure S12. Graphical representations of molecule A important physico-chemical features—Bioavailability Radar. The pink area represents the optimal range for each properties (lipophilicity: XLOGP3 between −0.7 and +5.0, size: MW between 150 and 500 g/mol, polarity: TPSA between 20 and 130 Å2, solubility: log S not higher than 6, saturation: fraction of carbons in the sp3 hybridization not less than 0.25, and flexibility: no more than 9 rotatable bonds; Figure S13. Graphical representations of molecule A1 important physico-chemical features—Bioavailability Radar. The pink area represents the optimal range for each properties (lipophilicity: XLOGP3 between −0.7 and +5.0, size: MW between 150 and 500 g/mol, polarity: TPSA between 20 and 130 Å2, solubility: log S not higher than 6, saturation: fraction of carbons in the sp3 hybridization not less than 0.25, and flexibility: no more than 9 rotatable bonds; Figure S14. Graphical representations of molecule A2 important physico-chemical features—Bioavailability Radar. The pink area represents the optimal range for each properties (lipophilicity: XLOGP3 between −0.7 and +5.0, size: MW between 150 and 500 g/mol, polarity: TPSA between 20 and 130 Å2, solubility: log S not higher than 6, saturation: fraction of carbons in the sp3 hybridization not less than 0.25, and flexibility: no more than 9 rotatable bonds; Figure S15. Graphical representations of molecule A3 important physico-chemical features—Bioavailability Radar. The pink area represents the optimal range for each properties (lipophilicity: XLOGP3 between −0.7 and +5.0, size: MW between 150 and 500 g/mol, polarity: TPSA between 20 and 130 Å2, solubility: log S not higher than 6, saturation: fraction of carbons in the sp3 hybridization not less than 0.25, and flexibility: no more than 9 rotatable bonds; Figure S16. Graphical representations of molecule A4 important physico-chemical features—Bioavailability Radar. The pink area represents the optimal range for each properties (lipophilicity: XLOGP3 between −0.7 and +5.0, size: MW between 150 and 500 g/mol, polarity: TPSA between 20 and 130 Å2, solubility: log S not higher than 6, saturation: fraction of carbons in the sp3 hybridization not less than 0.25, and flexibility: no more than 9 rotatable bonds; Figure S17. Graphical representations of molecule A5 important physico-chemical features—Bioavailability Radar. The pink area represents the optimal range for each properties (lipophilicity: XLOGP3 between −0.7 and +5.0, size: MW between 150 and 500 g/mol, polarity: TPSA between 20 and 130 Å2, solubility: log S not higher than 6, saturation: fraction of carbons in the sp3 hybridization not less than 0.25, and flexibility: no more than 9 rotatable bonds; Figure S18. Graphical representations of molecule A6 important physico-chemical features—Bioavailability Radar. The pink area represents the optimal range for each properties (lipophilicity: XLOGP3 between −0.7 and +5.0, size: MW between 150 and 500 g/mol, polarity: TPSA between 20 and 130 Å2, solubility: log S not higher than 6, saturation: fraction of carbons in the sp3 hybridization not less than 0.25, and flexibility: no more than 9 rotatable bonds; Figure S19. Graphical representations of molecule A7 important physico-chemical features—Bioavailability Radar. The pink area represents the optimal range for each properties (lipophilicity: XLOGP3 between −0.7 and +5.0, size: MW between 150 and 500 g/mol, polarity: TPSA between 20 and 130 Å2, solubility: log S not higher than 6, saturation: fraction of carbons in the sp3 hybridization not less than 0.25, and flexibility: no more than 9 rotatable bonds; Figure S20. Graphical representations of molecule A8 important physico-chemical features—Bioavailability Radar. The pink area represents the optimal range for each properties (lipophilicity: XLOGP3 between −0.7 and +5.0, size: MW between 150 and 500 g/mol, polarity: TPSA between 20 and 130 Å2, solubility: log S not higher than 6, saturation: fraction of carbons in the sp3 hybridization not less than 0.25, and flexibility: no more than 9 rotatable bonds; Figure S21. Graphical representations of molecule A9 important physico-chemical features—Bioavailability Radar. The pink area represents the optimal range for each properties (lipophilicity: XLOGP3 between −0.7 and +5.0, size: MW between 150 and 500 g/mol, polarity: TPSA between 20 and 130 Å2, solubility: log S not higher than 6, saturation: fraction of carbons in the sp3 hybridization not less than 0.25, and flexibility: no more than 9 rotatable bonds.
