# Supplementary material for: Development of Novel Therapeutics for Schizophrenia Treatment Based on a Selective Positive Allosteric Modulation of α1-Containing GABAARs—In Silico Approach
Source: Curr Issues Mol Biol. 2022 Jul 29;44(8):3398–412. doi: 10.3390/cimb44080234 (PMC9406691; doi:10.3390/cimb44080234)
Supplement: Supplementary file 1 [file cimb-44-00234-s001.zip › cimb-1803194-supplementary.pdf]

Supplementary Materials

Development of Novel Therapeutics for Schizophrenia Treatment Based on a Selective Positive Allosteric Modulation of α1-Containing GABAARs—In Silico Approach

Table of content

The Monte Carlo optimization method……………………………………………… 1

Table S1……………………………………………………………………………… 4

Table S2……………………………………………………………………………… 6

Table S3……………………………………………………………………………… 7

Figure S1 …………………………………………………………………………….8

Table S4……………………………………………………………………………… 9

Figures S2-S11……………………………………………………………………….11

Table S5……………………………………………………………………………… 21

Figures S12-S21…………………………………………………………………………25

The Monte Carlo optimization method

Each optimal descriptor which is obtained by utilizing the Monte Carlo method is assigned a numerical value, i.e. the correlation weight (CW). This process is resolved by the Monte Carlo method through the creation of appropriate random numbers and observation of the manner in which this number fraction obeys a property or certain properties, where the CW value is randomly assigned to descriptors based on the SMILES in each independent Monte Carlo run and for a defined endpoint. Numerical data calculations concerning the correlation weights that yield the maximum correlation coefficient value between an endpoint and the optimal descriptor are achieved by employing the optimization process of the Monte Carlo method. Two parameters – Threshold (T) and the Number of epochs (N_{epoch}) should be taken into account when this method is utilized for the purpose of building a QSAR model. Threshold represents a coefficient for the classification of numerous molecular features (including both SMILES based molecular fragments and SMILES based indices), calculated from the SMILES notation and classified into two categories: a) active (in this case, the modeling process involves the correlation weight); and b) rare (the modeling process does not include the correlation weight in this case). The process is realized as follows: the molecule descriptor X should be removed from model building in case a single molecular feature (X) defined from the SMILES notation of molecules within the training set occurs less than T times. Thus, the numerical value representing this feature (the correlation weight of the X,

CW(X)) is set as zero, and is, therefore, defined as rare. All the other molecular features can be used for model building and are active. The training set's best statistical quality is provided by N_{epoch}, which signifies the Monte Carlo optimization epoch number. Once an unlimited epoch number is applied, the above-mentioned Monte Carlo optimization provides the maximum correlation coefficient for the training set. Nevertheless, the maximum correlation coefficient between the optimal descriptor and the endpoint for the external test set exists for a certain number of epochs. Calculations favor this epoch number, as there is good predictive potential of the obtained model, in the event that the number of epochs reaches the said value. Conversely, the T increase is followed by the training set's correlation coefficient decrease. Nonetheless, the threshold providing the test set's maximum correlation coefficient does exist. The mentioned threshold is preferable when observed from the practical perspective. Preferable values of the threshold, as well as the Monte Carlo optimization epoch number (T and N_{epoch}), need to be defined for the purpose of preparing a proper QSAR model with the application of the both SMILES notation and molecular-graph based optimal descriptor. Monte Carlo method simulations are run on the basis of iterative algorithms, with the aim of revealing the distribution of an unknown probabilistic entity. The Monte Carlo optimization process does include a number of epochs of the training set for a specified target function. The first operation is the termination of CW (SA) for each SMILES attribute SA, with the starting values of all CWs set to $1 \pm 0.01 \times$Rnd (Rnd stands for the random value generator,

with a range of 0 to 1). A random sequence replaces the regular attribute number order. The following step involves the assessment of the target function's starting value, as well as further correlation weight modification. Afterwards, relevant steps are repeated in the Monte Carlo optimization for all the non-rare attributes. The QSAR model is calculated (by using the training set) through the utilization of the linear regression approach (Eq. 1), once the numerical data regarding the correlation weights from the model is obtained, with preferable statistics for the test set. In the presented research the search for the best combination of T and N_{epoch} was performed within values 1-5 for T and 0-50 for N_{epoch}.

$$Ac = C_0 + C_1 \times DCW(T, N_{epoch}) \quad (1)$$

which are already in use in QSAR model development. The computations were conducted with the CORAL software, and the use of Eq. 2

$$d(A) = \frac{|P(A_{train}) - P(A_{test})|}{N(A_{train}) - N(A_{trest})} \quad (2)$$

In the Eq, $P(A_{train})$ and $P(A_{calib})$ define the probabilities of a conformation-independent attribute or descriptor (A) in the training and test sets, respectively, while $N(A_{train})$ and $N(A_{calib})$ define the frequency of a conformation-independent attribute or descriptor (A) which appears in the training and test sets, respectively. The statistical SMILES defect (D) is the sum of defects, d(A), of all the attributes available in the molecules SMILES notation, and it is mathematically defined as in Eq. 3

$$D = defect(SMILES) = \sum_{k=1}^{NA} d(A) \quad (3)$$

The molecule is not classified in defined AD, and is a categorized outlier, if its $D > 2 \times D_{av}$; where D_{av} is the average of the D calculated for the appropriate set (training or test) where the molecule is placed.

Table S1. The SMILES notation of the studied molecules, calculated values for the DCW, experimental data (Ac) – expr, the values of Ac calculated with the application of CORAL software – calc, the difference between expr and calc – diff for the built QSPR model.

SMILES notation		Expr.	Split 1				Split 2				Split 3			
			DCW	Calc.	Diff	Set	DCW	Calc.	Diff	Set	DCW	Calc.	Diff	Set
1	Fc1ccc(cc1)c1nc2n(c1CC(=O)N(C)C)cc(cc2)C	7.222	134.4098	7.1681	0.0539	Tr	151.751	7.042	0.18	Tr	108.9424	7.0892	0.1328	Ts
2	Cc1ccc(cc1)c1nc2n(c1CC(=O)N(C)C)cc(cc2)F	7.602	137.6367	7.3656	0.2364	Ts	161.577	7.4486	0.1534	Tr	113.4444	7.516	0.086	Tr
3	Fc1ccc2n(c1)c(CC(=O)N(C)C)c(n2)c1ccccc1	6.886	125.1984	6.6043	0.2817	Tr	155.6749	7.2043	-0.3183	Tr	105.4278	6.7559	0.1301	Tr
4	Fc1cccc(c1)c1nc2n(c1CC(=O)N(C)C)cc(cc2)F	6.854	128.7993	6.8247	0.0293	Tr	145.1153	6.7674	0.0866	Tr	106.8432	6.8901	-0.0361	Ts
5	Fc1ccc(c(c1)F)c1nc2n(c1CC(=O)N(C)C)cc(cc2)F	6.745	130.4678	6.9269	-0.1819	Tr	144.1057	6.7256	0.0194	Tr	106.9761	6.9027	-0.1577	Tr
6	Fc1ccc2n(c1)c(CC(=O)N(C)C)c(n2)c1ccc(cc1)C(F)(F)F	7.167	135.961	7.2631	-0.0961	Tr	157.2942	7.2713	-0.1043	Ts	109.9812	7.1877	-0.0207	Tr
7	Fc1ccc(cc1)c1nc2n(c1CC(=O)N(C)C)cc(cc2)C(F)(F)F	6.292	123.871	6.5231	-0.2311	Tr	138.9836	6.5137	-0.2217	Tr	101.4781	6.3814	-0.0894	Tr
8	Fc1ccc(c(c1)F)c1nc2n(c1CC(=O)N(C)C)cc(cc2)C(F)(F)F	6.081	118.1072	6.1703	-0.0893	Ts	128.9192	6.0972	-0.0162	Tr	99.48486	6.1924	-0.1114	Tr
9	O=C(N(C)C)Cc1c(nc2n1cc(cc2)C(F)(F)F)c1ccc(cc1)C(F)(F)F	6.312	119.9693	6.2843	0.0277	Tr	134.3022	6.3199	-0.0079	Tr	100.4002	6.2792	0.0328	Ts
10	Fc1ccc(cc1)c1nc2n(c1CC(=O)N(C)C)cc(cc2)F	7.252	136.2317	7.2796	-0.0276	Tr	154.1701	7.1421	0.1099	Tr	108.9694	7.0917	0.1603	Tr
11	Fc1ccc2n(c1)c(CC(=O)N(C)C)c(n2)c1ccc(c(c1)F)F	7.237	134.0591	7.1467	0.0903	Tr	157.603	7.2841	-0.0471	Tr	111.9678	7.376	-0.139	Tr
12	Fc1cccc(c1)c1nc2n(c1CC(=O)N(C)C)cc(cc2)C(F)(F)F	6.137	116.4386	6.0682	0.0688	Tr	129.9288	6.139	-0.002	Ts	99.35189	6.1798	-0.0428	Tr
13	CC(=O)NCc1c(nc2n1cc(C)cc2)c1ccc(cc1)F	7.106	135.5063	7.2352	-0.1292	Tr	156.2188	7.2268	-0.1208	Tr	110.0986	7.1988	-0.0928	Tr
14	CCC(=O)NCc1c(nc2n1cc(C)cc2)c1ccc(cc1)F	7.565	139.1654	7.4592	0.1058	Tr	158.8357	7.3351	0.2299	Tr	111.9006	7.3697	0.1953	Tr
15	O=C(C(C)C)NCc1c(nc2n1cc(C)cc2)c1ccc(cc1)F	6.439	128.3742	6.7987	-0.3597	Tr	141.1235	6.6022	-0.1632	Tr	104.3177	6.6506	-0.2116	Ts
16	O=C(C1CC1)NCc1c(nc2n1cc(C)cc2)c1ccc(cc1)F	6.836	133.7281	7.1264	-0.2904	Tr	154.9261	7.1733	-0.3373	Ts	109.6711	7.1583	-0.3223	Tr

17	CC(=O)NCc1c(nc2n1cc(F)cc2)c1ccc(cc1)C	7.357	137.6209	7.3647	-0.0077	Tr	163.2778	7.5189	-0.1619	Tr	112.9383	7.4681	-0.1111	Tr
18	CCC(=O)NCc1c(nc2n1cc(F)cc2)c1ccc(cc1)C	7.438	141.28	7.5886	-0.1506	Ts	165.8947	7.6272	-0.1892	Tr	114.7403	7.6389	-0.2009	Tr
19	O=C(C(C)C)NCc1c(nc2n1cc(F)cc2)c1ccc(cc1)C	7.058	130.4888	6.9281	0.1299	Tr	148.1825	6.8943	0.1637	Tr	107.1574	6.9199	0.1381	Tr
20	O=C(C1CC1)NCc1c(nc2n1cc(F)cc2)c1ccc(cc1)C	7.259	135.8427	7.2558	0.0032	Ts	161.9851	7.4654	-0.2064	Ts	112.5108	7.4275	-0.1685	Tr
21	CC(=O)NCc1c(nc2n1cc(F)cc2)c1ccc(cc1)F	7.367	136.2457	7.2805	0.0865	Tr	160.0313	7.3846	-0.0176	Tr	110.8253	7.2677	0.0993	Tr
22	CCC(=O)NCc1c(nc2n1cc(F)cc2)c1ccc(cc1)F	7.432	139.9049	7.5045	-0.0725	Tr	162.6483	7.4929	-0.0609	Tr	112.6274	7.4386	-0.0066	Ts
23	O=C(C(C)C)NCc1c(nc2n1cc(F)cc2)c1ccc(cc1)F	6.51	129.1137	6.844	-0.334	Ts	144.9361	6.76	-0.25	Tr	105.0444	6.7195	-0.2095	Tr
24	O=C(C1CC1)NCc1c(nc2n1cc(F)cc2)c1ccc(cc1)F	7.68	134.4676	7.1717	0.5083	Tr	158.7386	7.3311	0.3489	Tr	110.3978	7.2272	0.4528	Tr
25	CC(=O)NCc1c(nc2n1cc(F)cc2)c1ccc(c(c1)F)F	7.409	137.9655	7.3858	0.0232	Tr	162.5801	7.4901	-0.0811	Ts	111.3877	7.321	0.088	Ts
26	CCC(=O)NCc1c(nc2n1cc(F)cc2)c1ccc(c(c1)F)F	7.62	141.6246	7.6097	0.0103	Tr	165.197	7.5983	0.0217	Tr	113.1897	7.4919	0.1281	Tr
27	Fc1ccc2n(c1)c(CNC(=O)C(C)C)c(n2)c1ccc(c(c1)F)F	7.143	135.7643	7.251	-0.108	Ts	152.416	7.0695	0.0735	Ts	109.2282	7.1163	0.0267	Ts
28	Fc1ccc2n(c1)c(CNC(=O)C1CC1)c(n2)c1ccc(c(c1)F)F	7.642	141.5832	7.6072	0.0348	Tr	165.5352	7.6123	0.0297	Tr	114.5703	7.6228	0.0192	Tr
29	CC(=O)NCc1c(nc2n1cc(F)cc2)c1ccc(cc1)C(F)(F)F	7.203	136.2854	7.2829	-0.0799	Ts	157.9488	7.2984	-0.0954	Ts	110.4185	7.2291	-0.0261	Ts
30	CCC(=O)NCc1c(nc2n1cc(F)cc2)c1ccc(cc1)C(F)(F)F	7.319	139.9445	7.5069	-0.1879	Tr	160.5657	7.4067	-0.0877	Tr	112.2206	7.4	-0.081	Tr
31	O=C(C(C)C)NCc1c(nc2n1cc(F)cc2)c1ccc(cc1)C(F)(F)F	7.064	129.1533	6.8464	0.2176	Ts	142.8535	6.6738	0.3902	Tr	104.6376	6.681	0.383	Tr
32	O=C(C1CC1)NCc1c(nc2n1cc(F)cc2)c1ccc(cc1)C(F)(F)F	7.174	134.5072	7.1741	-0.0001	Tr	156.656	7.2449	-0.0709	Tr	109.991	7.1886	-0.0146	Tr
33	Cc1ccc(cc1)c1nc2n(c1CC(=O)N(C)C)cc(cc2)C	7.357	135.3153	7.2235	0.1335	Tr	154.4871	7.1552	0.2018	Ts	112.0902	7.3876	-0.0306	Tr

Table S2. Y-randomization of the best QSAR model (best optimization run) for three independent splits.

	Split 1		Split 2		Split 3	
Run	Training	Test	Training	Test	Training	Test
0	0.8302	0.8586	0.8518	0.8523	0.8411	0.9479
1	0.0164	0.0089	0.0785	0.0777	0.0012	0.5381
2	0.0085	0.0275	0.0912	0	0.0002	0.0336
3	0.0015	0.1233	0.0155	0.1729	0.0149	0.3295
4	0.0855	0.0517	0.0137	0.2325	0.0095	0.0983
5	0.0205	0.096	0.0011	0.3956	0.0173	0.4075
6	0.0033	0.1001	0.0592	0.0834	0.0471	0.3418
7	0.0024	0.0324	0.1329	0.6299	0.0141	0.0583
8	0.0221	0.2024	0.016	0.0599	0.432	0.3368
9	0.0329	0.3063	0.0613	0.1237	0.0759	0.0203
10	0.0426	0.1857	0.065	0.2873	0.0195	0.0008
R_r^2	0.0236	0.1134	0.0534	0.2063	0.0632	0.2165
${}^{C}R_p^2$	0.8184	0.7999	0.8246	0.742	0.8089	0.8327
${}^{C}R_p^2 = R \times (R^2 - R_r^2)^{1/2}$ should be > 0.5						

Table S3. The statistical quality of QSAR models developed with the GA-MLR method for selective positive allosteric modulation of α1-containing $GABA_A$Rs.

Fitting criteria									
R^2	R^2_{adj}	R^2-R^2_{adj}	LOF	$RMSE_{tr}$	MAE_{tr}	RSS_{tr}	CCC_{tr}	s	F
0.8986	0.8719	0.0267	0.0566	0.1427	0.1208	0.5092	0.9466	0.1637	33.6638
Internal validation criteria									
Q^2_{loo}	R^2-Q^2_{loo}	$RMSE_{cv}$	MAE_{cv}	$PRESS_{cv}$	CCC_{cv}				
0.8410	0.0576	0.1787	0.1551	0.7983	0.9171				
External validation criteria									
$RMSE_{ext}$	MAE_{ext}	$PRESS_{ext}$	R^2_{ext}	R^2_m(av)	ΔR^2_m	CCC_{extr}			
0.2609	0.2259	0.5448	0.6303	0.6453	0.1780	0.7778			

R^2 – The Coefficient of Determination; R^2_{adj} – adjusted R^2
LOF – Lack-of-fit
LOO – Leave One Out
RMSE – Root Mean Square Error; $RMSE_{tr}$ – for training set, $RMSE_{cv}$ – for internal validation; $RMSE_{ext}$ – for external validation
MAE – Mean Absolute Error; MAE_{tr} – for training set, MAE_{cv} – for internal validation
RSS – Residual Sum of Squares
CCC – The Concordance Correlation Coefficient; CCC_{tr} – for training set, CCC_{cv} – for internal validation, CCC_{extr} – for external validation
s – Standard Deviation
Sp – Split
PRESS – The Predicted Residual Error Sum of Squares; $PRESS_{cv}$ – for internal validation; $PRESS_{ext}$ – for external validation
R^2_m, R^2_m(av), ΔR^2_m – [49,50]

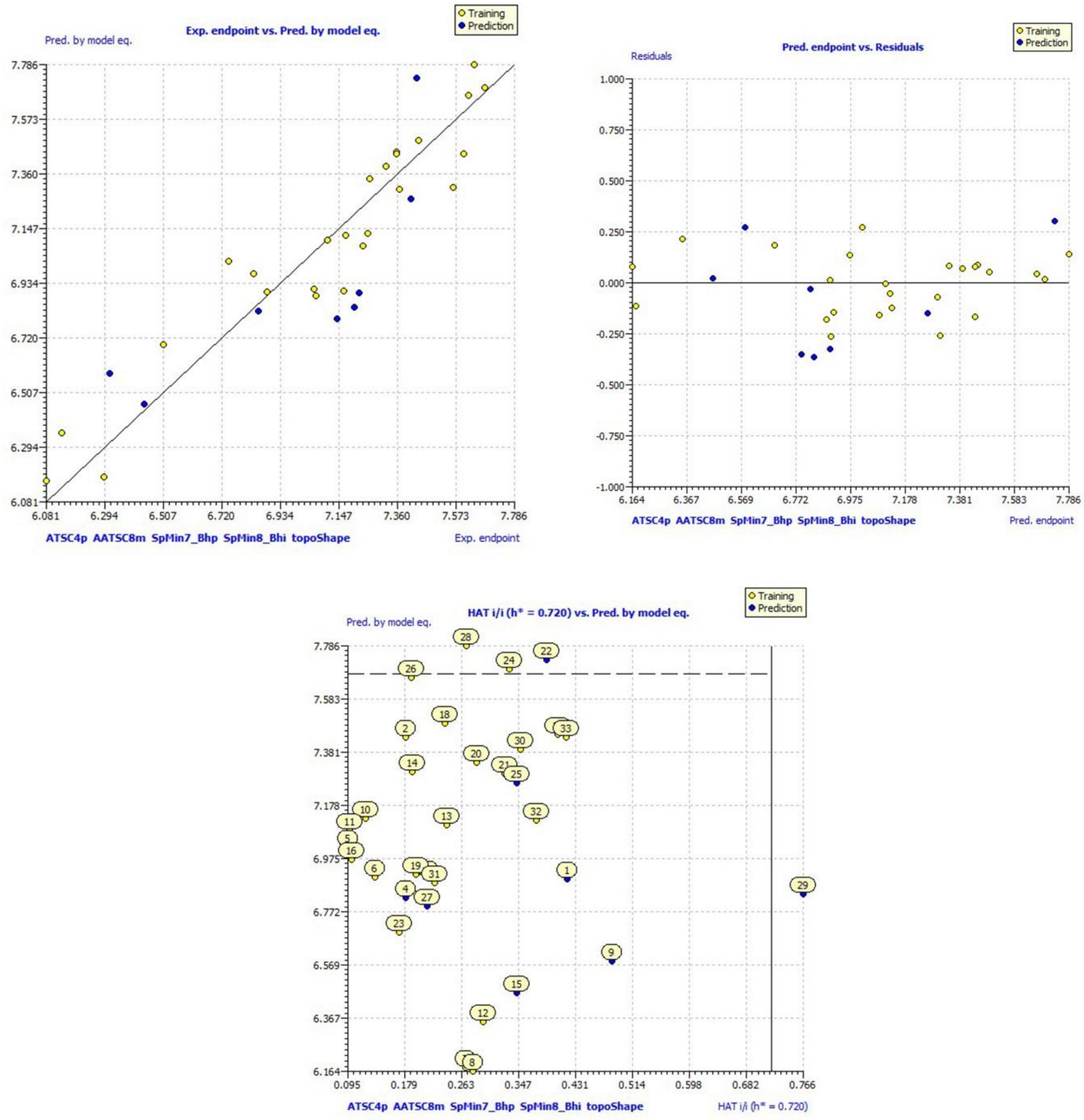

Figure S1. Above left) Graphical representation of developed QSAR model with GA-MLR method for split 3; Above right) Difference between experimental and calculated pKi values; Bellow) Graphical representation of applicability domain established for split 3.

Table S4. The list of SAks together with their correlation weights for the three runs of the Monte Carlo optimization.

SA	CW(SA)			SA	CW(SA)			SA	CW(SA)			SA	CW(SA)		
	Run 1	Run 2	Run 3		Run 1	Run 2	Run 3		Run 1	Run 2	Run 3		Run 1	Run 2	Run 3
10011001000	0.35642	0.23382	0.26157	EC0-C...2...	0.06835	0.27859	0.09176	P4E0F...1-..	0.57325	0.36197	0.00238	S3E0C...6-..	0.42114	0.09365	1.42081
(...(.......	-0.69934	-0.84196	-0.86727	EC0-C...3...	0.08216	0.32373	-0.12695	P4E0F...3-..	0.17643	-0.5171	0.17985	S3E0C...7-..	-0.06135	-0.03479	0.45387
(..........	-0.96877	0.13154	0.03524	EC0-C...4...	0.32932	0.1743	0.33224	P4E0F...4-..	0.15862	0.34736	0.00274	S3E0C...8-..	1.42208	0.08211	-0.24504
(...C...(...	-0.56963	-0.23779	0.11164	EC0-F...1...	0.31893	0.03891	0.35164	P4E0N...11-.	-0.39943	-0.15051	0.29021	S3E0C...9-..	0.27386	0.71778	0.45792
(...F...(...	0.13968	0.40324	0.39255	EC0-N...2...	-0.89611	-0.10668	0.21018	P4E0N...2-..	0.47432	0.41226	0.46791	S3E0F...3-..	0.0182	0.21118	0.14754
(...N...(...	0.08117	-0.20043	0.37089	EC0-N...3...	0.38623	0.37009	0.45651	P4E0N...3-..	0.22047	0.25224	0.44858	S3E0F...4-..	0.6034	0.12524	0.2638
(...c...(...	2.22696	0.6362	0.56735	EC0-O...1...	3.57941	1.29558	0.57899	P4E0N...6-..	0.30207	0.15962	0.34477	S3E0F...5-..	0.52848	0.07988	0.35287
++++F---B2==	-0.95197	-0.78488	0.11804	Cmax.2......	0.84673	-0.74003	-0.31991	P4E0N...8-..	0.07336	0.13161	0.69344	S3E0N...0-..	0.7391	0.27477	0.9025
++++F---N===	0.68268	-0.55718	0.46757	HALO10000000	0.53343	-0.74754	-0.57033	P4E0O...0-..	0.057	2.19436	0.11251	S3E0N...12-.	0.05738	0.39593	-0.08657
++++F---O===	0.70662	0.26801	0.68831	N...(.......	0.05753	-0.87819	0.04487	P4E0O...1-..	0.42126	-0.77726	2.16569	S3E0N...13-.	2.28487	0.47296	0.34752
++++N---B2==	1.49587	1.68384	0.1278	N...(...1...	1.32681	0.36892	0.22079	P4E0O...4-..	0.31017	0.45249	0.36752	S3E0N...14-.	0.03332	2.11362	0.04791
++++N---O===	1.26051	-0.61814	-0.10939	N...(...C...	1.04832	0.22157	-0.9545	NNE0C...100-	-0.64284	0.46327	0.28993	S3E0N...16-.	-0.8916	0.11312	-0.10556
++++O---B2==	0.21525	1.34551	0.28271	N..........	-0.04657	0.03809	0.62252	NNE0C...109-	1.17197	1.31417	0.11364	S3E0N...4-..	0.25972	0.27004	1.24304
1...(.......	0.39138	0.08809	0.47401	N...C.......	0.07658	0.13319	0.15538	NNE0C...209-	-0.07674	0.3688	-0.11826	S3E0N...5-..	2.30113	0.15458	0.1524
1..........	0.05388	0.10762	0.12612	O...(.......	2.11717	0.19829	0.1525	NNE0C...218-	0.29355	1.4815	0.37006	S3E0N...6-..	0.15514	0.47705	0.35691
1...C...(...	0.49847	0.20937	0.2968	O...(...N...	-0.49479	2.3063	0.41785	NNE0C...309-	3.6671	0.86136	0.746	S3E0N...8-..	0.01286	0.07288	0.13773
1...c...(...	1.20547	0.2797	0.49381	O..........	-0.09234	1.85565	0.34035	NNE0C...318-	0.34766	1.3983	0.17262	S3E0O...1-..	0.30786	0.15226	0.48253
2...(.......	0.82073	-0.0558	2.1363	O...=...(...	0.6219	0.0374	0.24402	NNE0C...327-	0.14206	0.07528	-0.82729	S3E0O...2-..	0.34448	0.16025	0.49362
2..........	4.43025	1.04778	-0.39335	O...=.......	1.98294	0.26002	0.11311	NNE0C...409-	-0.08408	0.06737	0.46547	S3E0O...3-..	0.54006	0.07351	0.2679
2...n...(...	-0.78447	0.64603	0.26353	O...=...C...	0.17137	-0.85878	0.28021	NNE0F...109-	0.17104	0.04266	0.2175	S3E0O...4-..	0.4231	0.16047	0.05388
2...n...1...	0.01272	0.31054	0.09959	P2E0C...0-..	0.08126	-0.31499	-0.29861	NNE0N...218-	0.15127	-0.36304	0.87293	S3E0O...5-..	0.29197	0.46461	0.31694
=...(.......	2.19753	0.0667	2.25705	P2E0C...1-..	0.45085	0.17831	0.18451	NNE0N...327-	1.12277	1.33325	-0.07433	Nmax.1......	0.04448	0.33102	2.54719
=..........	1.15916	1.30845	1.0621	P2E0C...2-..	0.41904	0.49248	0.25811	NNE0O...109-	1.49314	0.29791	0.55741	Omax.1......	0.04719	0.57638	1.0932
=...C...(...	0.34704	-0.6723	0.40252	P2E0F...1-..	0.29529	0.47325	0.03052	NOSP11000000	0.05576	3.46448	0.04009	Smax.0......	2.74095	1.41092	0.25848
=...O...(...	2.06223	-0.41614	0.01346	P2E0F...2-..	0.13896	0.43325	0.10709	S2E0C...0-..	-0.61396	0.13923	0.30852	c...(.......	0.45368	0.42124	0.29755
C...(.......	0.21444	-0.99758	0.20839	P2E0N...1-..	1.74764	1.55486	2.48098	S2E0C...1-..	0.47025	-1.39869	-0.05404	c...(...1...	-0.19454	0.19985	0.23577
C...(...1...	0.10565	1.47484	0.06912	P2E0N...2-..	1.4666	0.15504	-0.20153	S2E0C...10-.	0.47835	-0.00342	0.29249	c...(...2...	0.37061	2.14944	0.27224
C...(...2...	0.18099	-0.56178	0.4894	P2E0O...1-..	0.20798	0.82551	-1.04639	S2E0C...2-..	0.32619	0.23884	0.25825	c...(...C...	-0.46174	0.2322	-0.43361
C...(...=...	1.221	2.09654	0.37596	P3E0C...0-..	0.06508	0.27198	0.45199	S2E0C...3-..	0.31522	1.76242	0.19784	c...(...F...	0.29454	0.14981	0.34182
C...(...C...	0.81828	0.06949	0.06015	P3E0C...1-..	0.01581	0.01397	0.09111	S2E0C...4-..	0.23593	0.4571	0.41564	c...(...c...	0.3523	-0.07719	0.28375
C..........	0.11595	0.11628	0.33512	P3E0C...2-..	0.2558	-0.5124	0.06587	S2E0C...5-..	0.31377	0.37784	0.25622	c..........	0.07358	1.38156	1.55114

C...1...(...	0.25821	0.52842	0.10767	P3E0C...3-..	0.09259	0.27636	0.30663	S2E0C...6-..	0.35862	2.18222	0.20959	c...1...(...	0.05688	-0.85527	-0.75725
C...1.......	2.27243	0.40898	0.15764	P3E0C...4-..	0.04194	0.3519	0.24459	S2E0C...7-..	0.46482	0.05107	0.26483	c...1.......	0.07527	0.31251	0.00899
C...1...C...	0.05744	-0.67912	1.45991	P3E0C...5-..	-0.56098	-0.74197	0.19802	S2E0C...8-..	0.15625	0.35305	-0.08459	c...1...C...	0.04804	0.01228	0.44426
C...=.......	0.08516	-0.6696	-0.99787	P3E0C...6-..	0.00703	-0.67232	0.06252	S2E0C...9-..	0.0809	0.16918	-0.46553	c...1...c...	0.49148	0.05285	0.26015
C...C...(...	0.05632	0.41033	0.25372	P3E0F...1-..	0.18912	0.06532	0.32588	S2E0F...3-..	0.46974	2.43427	0.14793	c...2...(...	0.50067	0.20955	0.40148
C...C.......	0.02848	0.71946	0.06079	P3E0F...2-..	0.15443	0.19172	0.30608	S2E0F...4-..	0.33784	-0.93142	-0.70104	c...2.......	-0.58122	0.12353	-0.60989
C...C...1...	0.48766	0.00189	0.02743	P3E0N...0-..	0.2693	0.15747	0.42546	S2E0N...0-..	0.09003	1.13662	0.24434	c...C.......	1.29384	0.5357	0.51677
C...C...C...	0.10981	0.51173	-0.70427	P3E0N...1-..	0.04254	-0.28797	0.24309	S2E0N...3-..	0.12065	-0.23727	0.34401	c...C...N...	0.13042	0.40246	0.19186
C...N...(...	0.22709	-0.45147	-0.36093	P3E0N...2-..	0.39199	0.13542	-0.6324	S2E0N...4-..	1.572	0.03204	0.23015	c...F.......	0.29308	-0.917	0.33253
C...c...1...	1.01399	0.29198	2.06871	P3E0N...4-..	-0.63613	-0.30558	0.39532	S2E0N...5-..	0.01559	0.13158	0.08442	c...c...(...	0.10978	0.15756	0.38781
C3......0...	0.35816	0.33401	0.34265	P3E0N...5-..	0.31946	0.70975	-0.72298	S2E0N...9-..	1.64463	2.98021	-1.02188	c...c.......	0.35915	-1.40403	0.33516
C3......1...	0.21109	0.37967	-0.67726	P3E0O...0-..	2.18431	0.22265	0.3951	S2E0O...2-..	0.05195	1.34238	0.22009	c...c...1...	1.42864	0.48708	2.46116
C4......0...	0.88675	2.35239	0.19317	P3E0O...1-..	0.4736	-0.00274	0.12487	S2E0O...3-..	0.21602	2.68812	-0.12635	c...c...2...	1.1064	1.83059	0.32924
C5...AH.1...	1.4407	1.16884	-0.58578	P3E0O...2-..	0.38671	1.10868	0.48846	S2E0O...4-..	-0.84462	0.08312	0.27694	c...c...c...	0.73194	0.1231	0.03075
C6...AH.2...	0.62069	0.79755	2.29229	P4E0C...0-..	0.23334	-0.50918	0.43347	S3E0C...0-..	0.23725	2.2	0.44356	c...n...(...	0.21933	0.11419	-0.483
C7......0...	0.44636	0.58315	-0.58661	P4E0C...1-..	0.15703	0.46162	0.08541	S3E0C...1-..	-0.94406	-0.51616	-0.11402	c...n...1...	-0.91501	0.15972	0.20265
BOND10000000	0.23157	0.22905	3.63799	P4E0C...10-.	0.07118	-0.79067	0.06161	S3E0C...10-.	0.42966	0.13324	-0.81085	n...(.......	0.54276	0.41577	0.23137
F...(...(...	0.17638	0.49309	0.42853	P4E0C...11-.	-0.5278	0.22381	0.28658	S3E0C...11-.	0.21899	-0.97581	-0.08425	n...(...c...	0.33348	0.44934	0.43534
F...(.......	0.12616	0.28559	0.04601	P4E0C...12-.	0.46511	0.18559	-0.60763	S3E0C...12-.	0.14139	-1.63927	0.33999	n..........	1.34394	3.47203	-0.23603
F...(...1...	0.34065	-0.00683	0.31122	P4E0C...16-.	0.05473	-0.79435	0.26642	S3E0C...13-.	0.25498	0.30074	1.18489	n...1.......	0.15952	0.13782	0.20157
F...(...2...	1.6148	2.04746	1.83798	P4E0C...2-..	0.30375	0.2152	-0.71191	S3E0C...14-.	0.14064	2.41008	0.14859	n...1...c...	0.89217	0.09732	-0.62111
F...(...C...	0.22404	0.14162	-0.69696	P4E0C...3-..	0.23043	0.40998	0.48976	S3E0C...15-.	1.04368	0.24378	-0.01545	n...2...(...	0.02087	0.03464	-0.53977
F...(...F...	0.06726	0.30888	0.31511	P4E0C...4-..	0.28178	0.09912	0.17243	S3E0C...2-..	-0.66937	0.04288	0.16639	n...2.......	0.27524	0.27284	0.05172
F..........	0.00339	0.33487	0.56728	P4E0C...6-..	-0.92812	0.39666	0.33317	S3E0C...3-..	0.30442	0.09547	0.2352	n...2...c...	0.34822	0.95933	2.33365
F...c...1...	-0.20052	-0.6753	0.10564	P4E0C...7-..	0.39169	2.49583	2.03993	S3E0C...4-..	-0.05147	0.04231	0.31898	n...c.......	0.14722	0.20698	0.42777
EC0-C...1...	2.20491	0.87997	0.28749	P4E0C...8-..	0.25273	0.40714	0.19701	S3E0C...5-..	0.29217	0.17515	0.01379	n...c...2...	0.32725	0.29946	0.19304

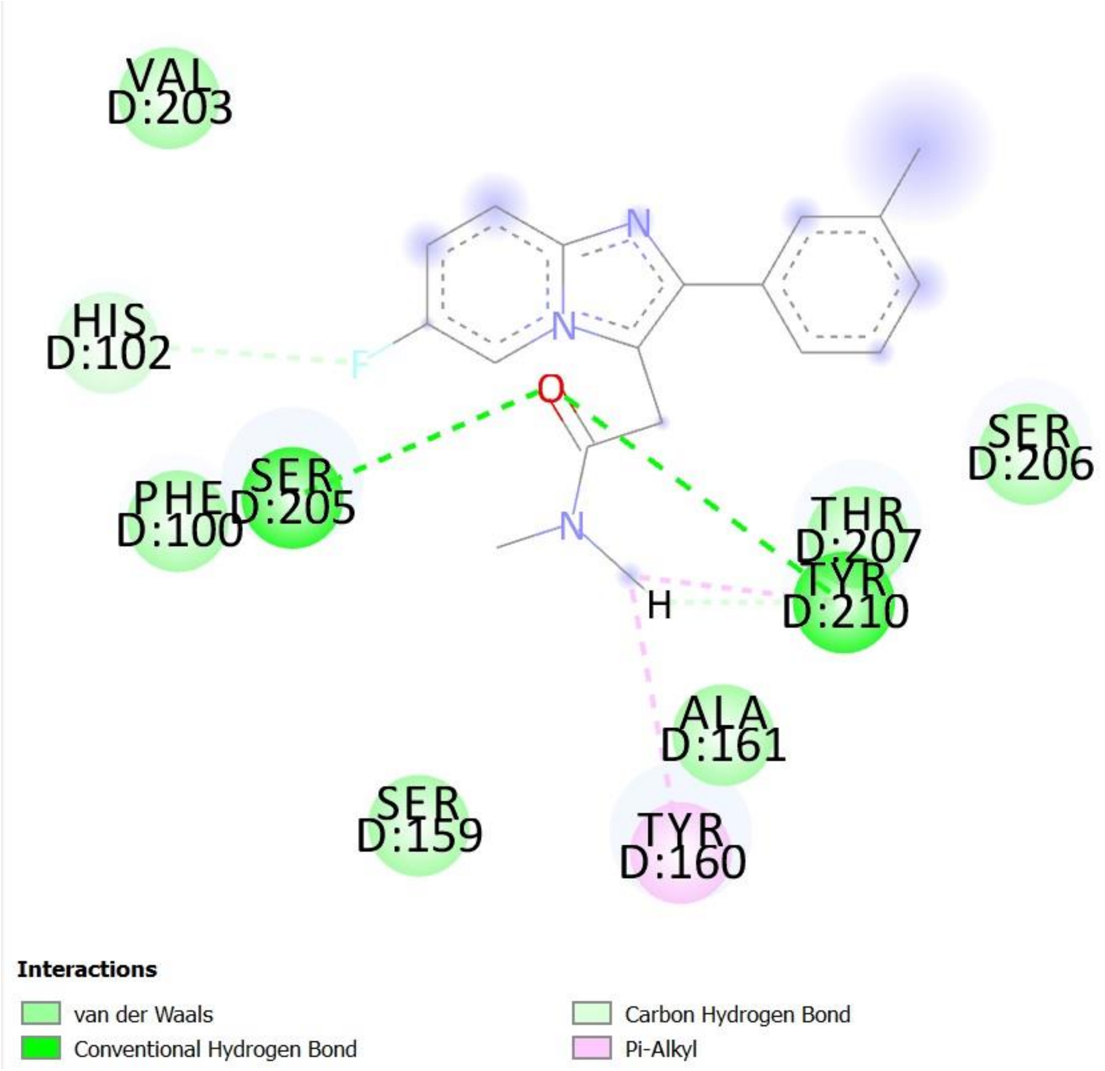

Figure S2. Two-dimensional representation of the interaction between molecule A and amino acids inside ionotropic GABA$_A$ receptor.

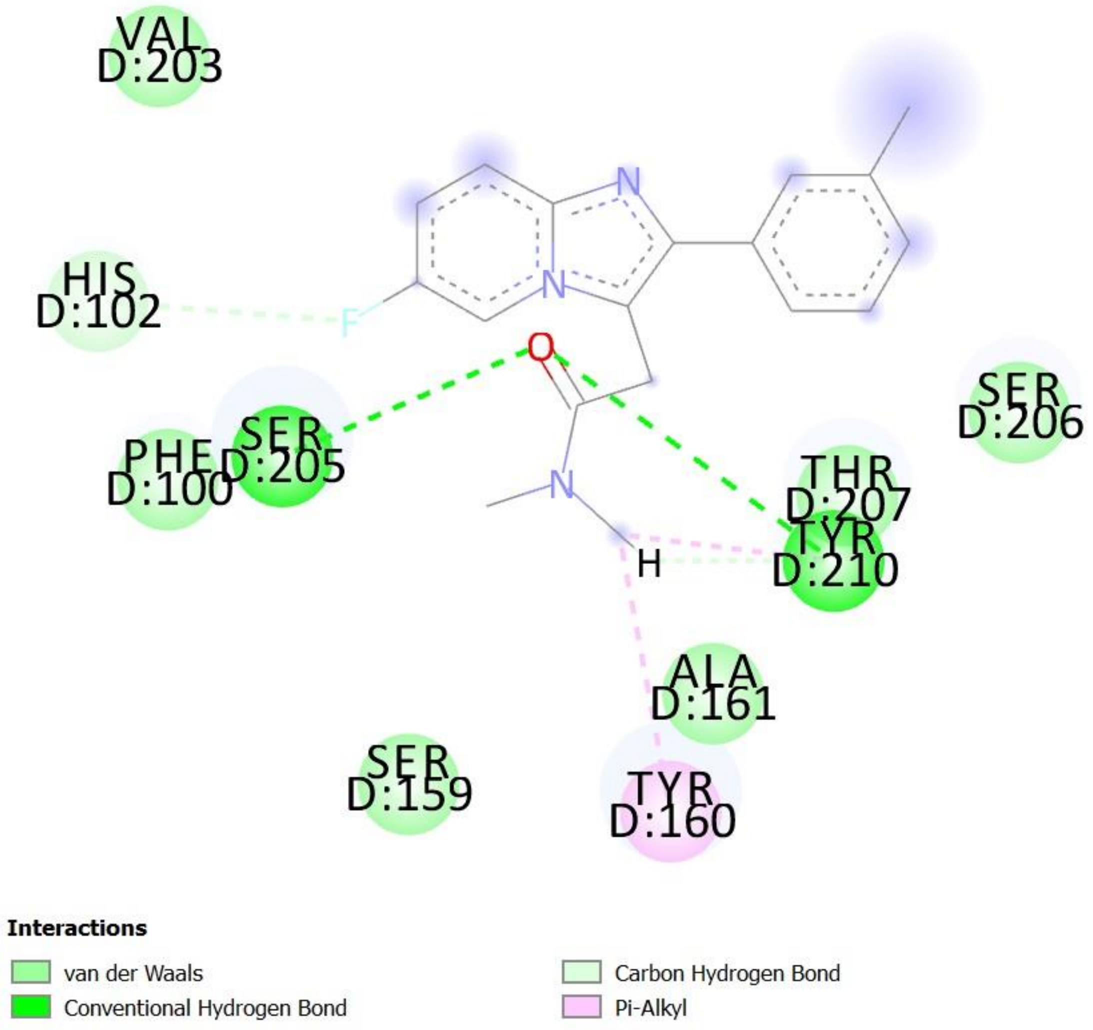

Figure S3. Two-dimensional representation of the interaction between molecule A1 and amino acids inside ionotropic $GABA_A$ receptor.

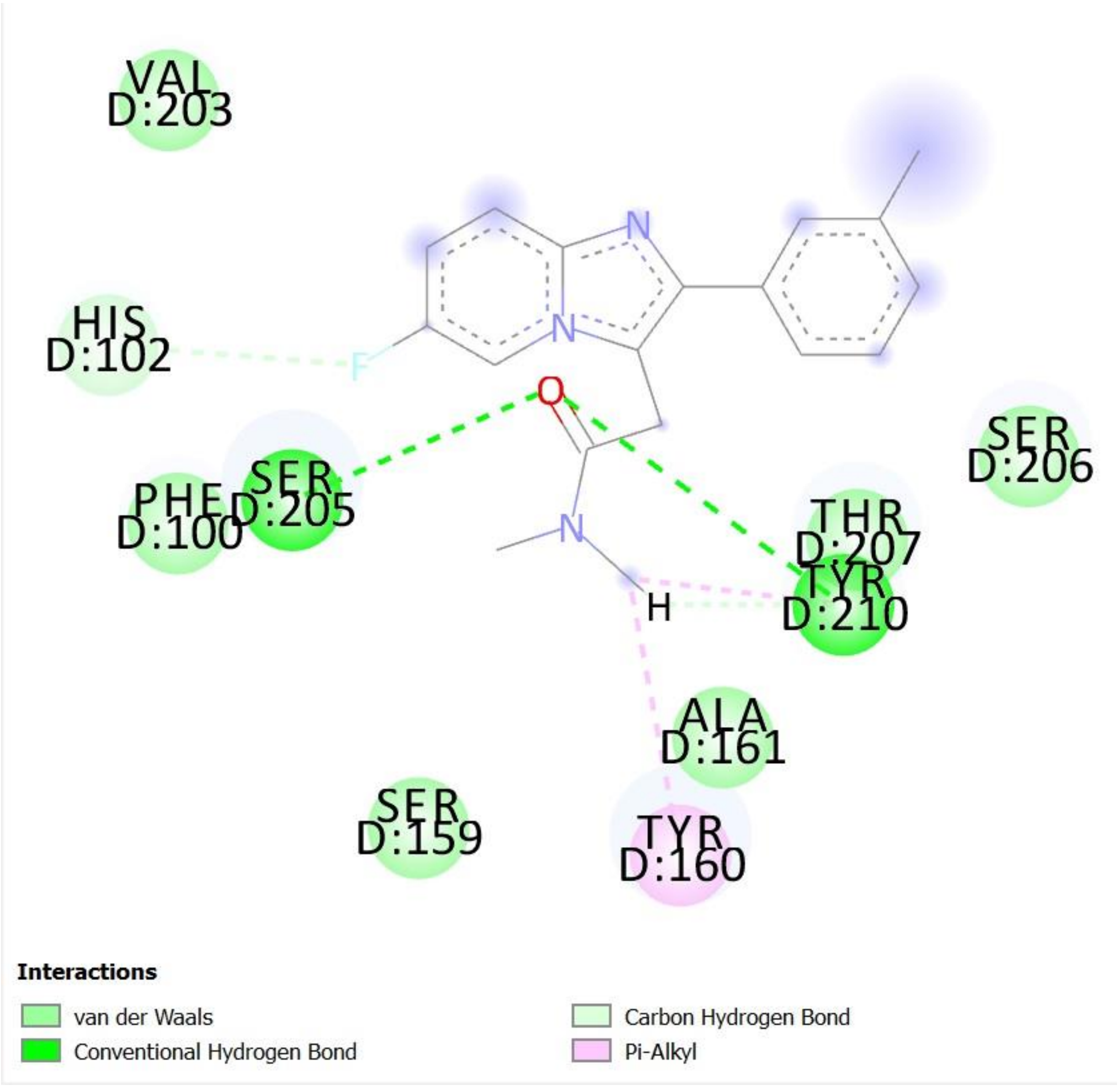

Figure S4. Two-dimensional representation of the interaction between molecule A2 and amino acids inside ionotropic $GABA_A$ receptor.

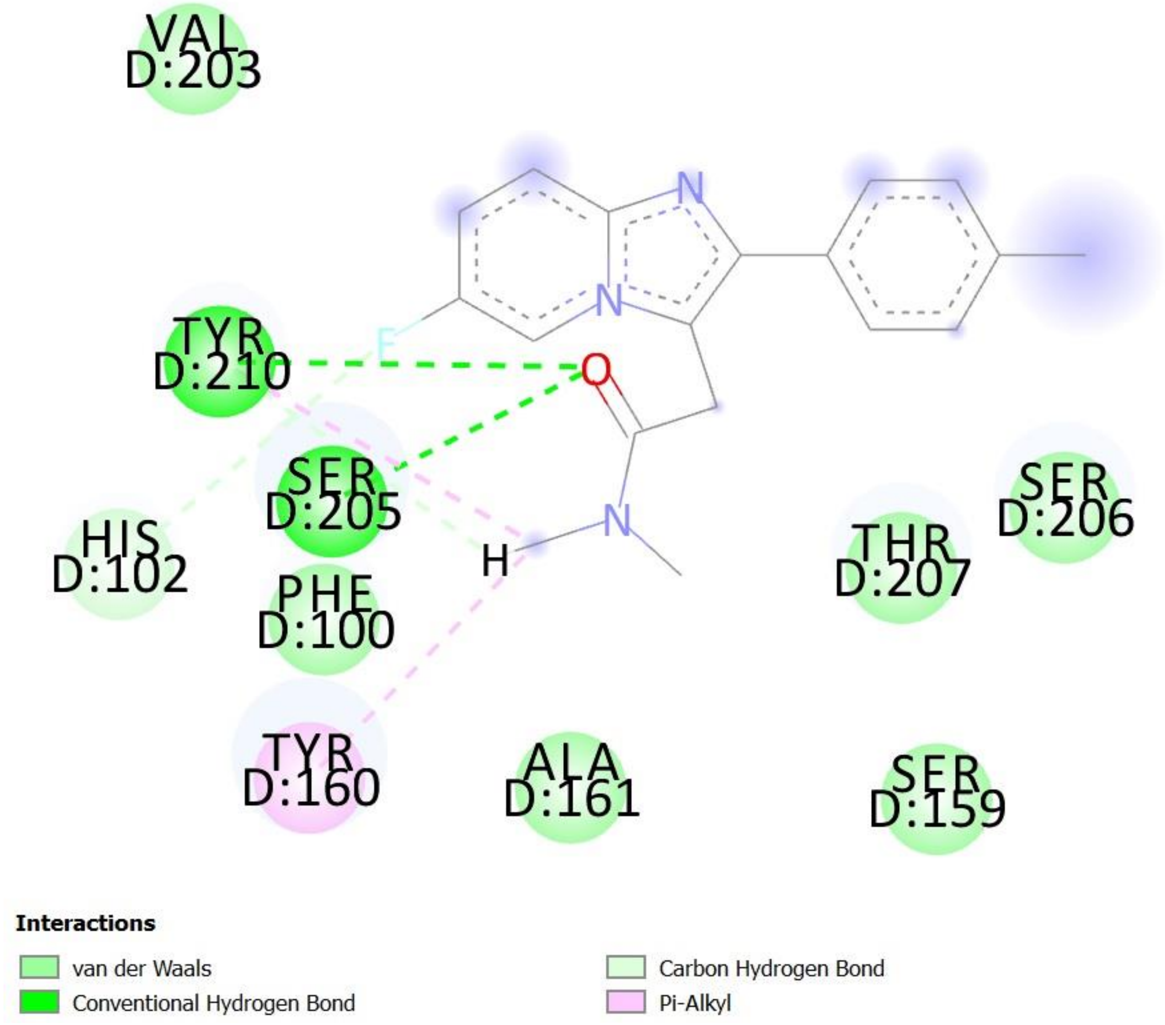

Figure S5. Two-dimensional representation of the interaction between molecule A3 and amino acids inside ionotropic $GABA_A$ receptor.

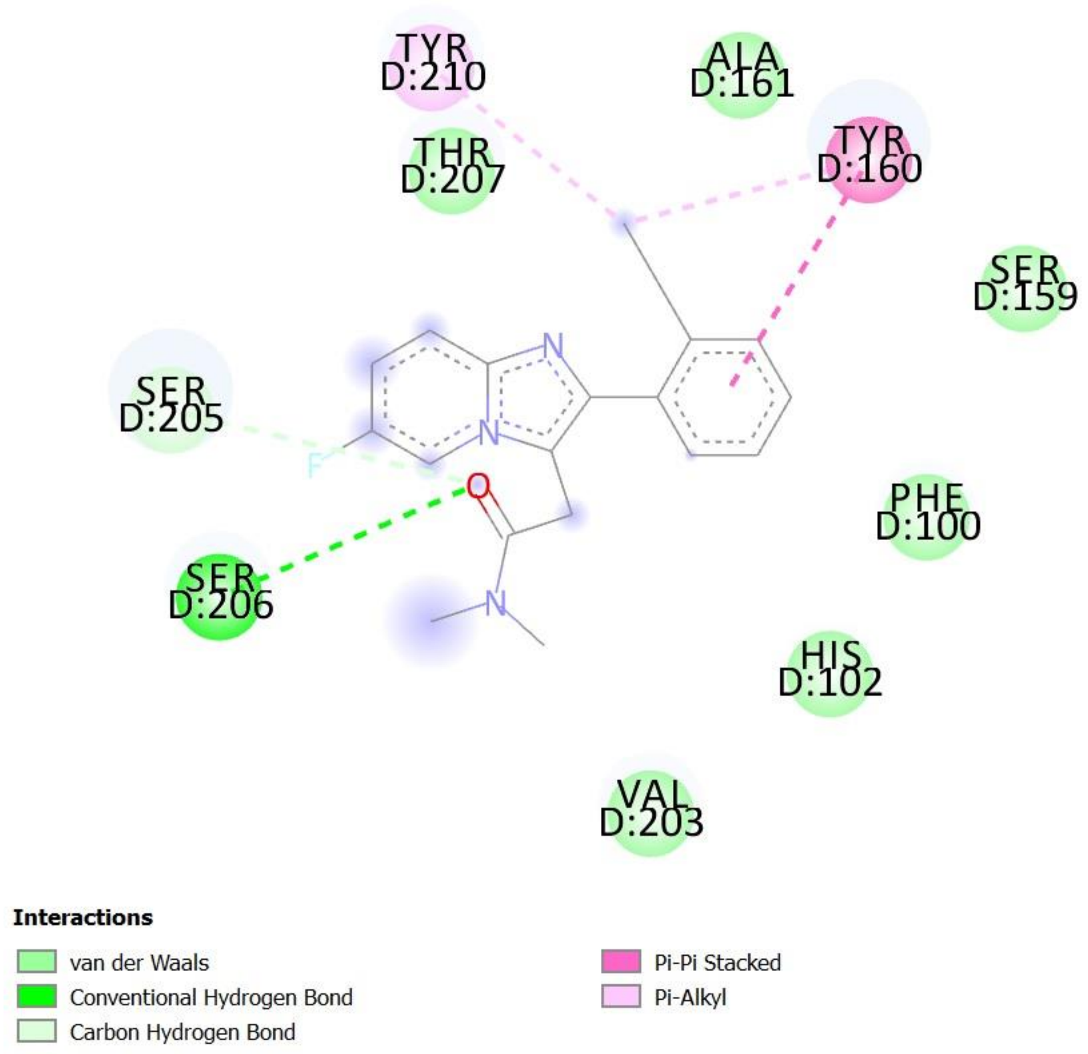

Figure S6. Two-dimensional representation of the interaction between molecule A4 and amino acids inside ionotropic $GABA_A$ receptor.

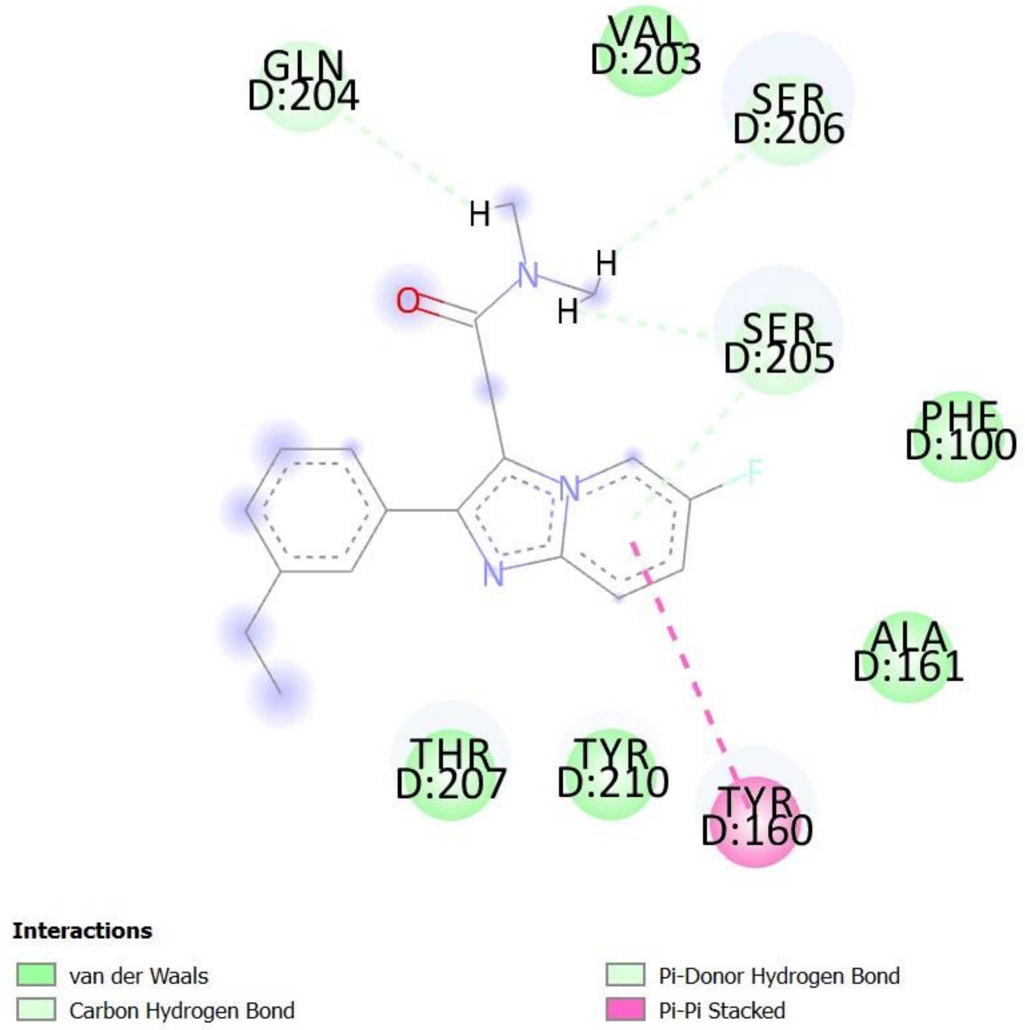

Figure S7. Two-dimensional representation of the interaction between molecule A5 and amino acids inside ionotropic $GABA_A$ receptor.

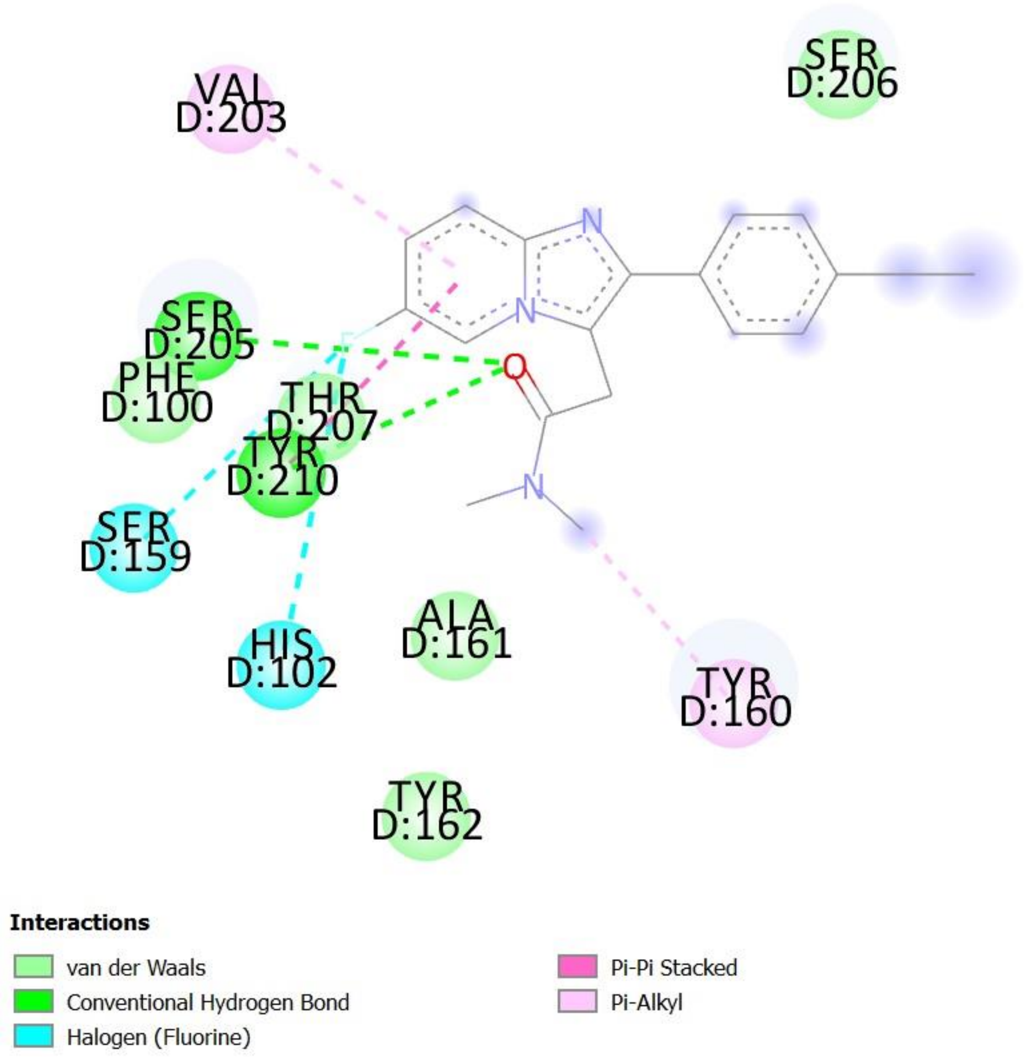

Figure S8. Two-dimensional representation of the interaction between molecule A6 and amino acids inside ionotropic $GABA_A$ receptor.

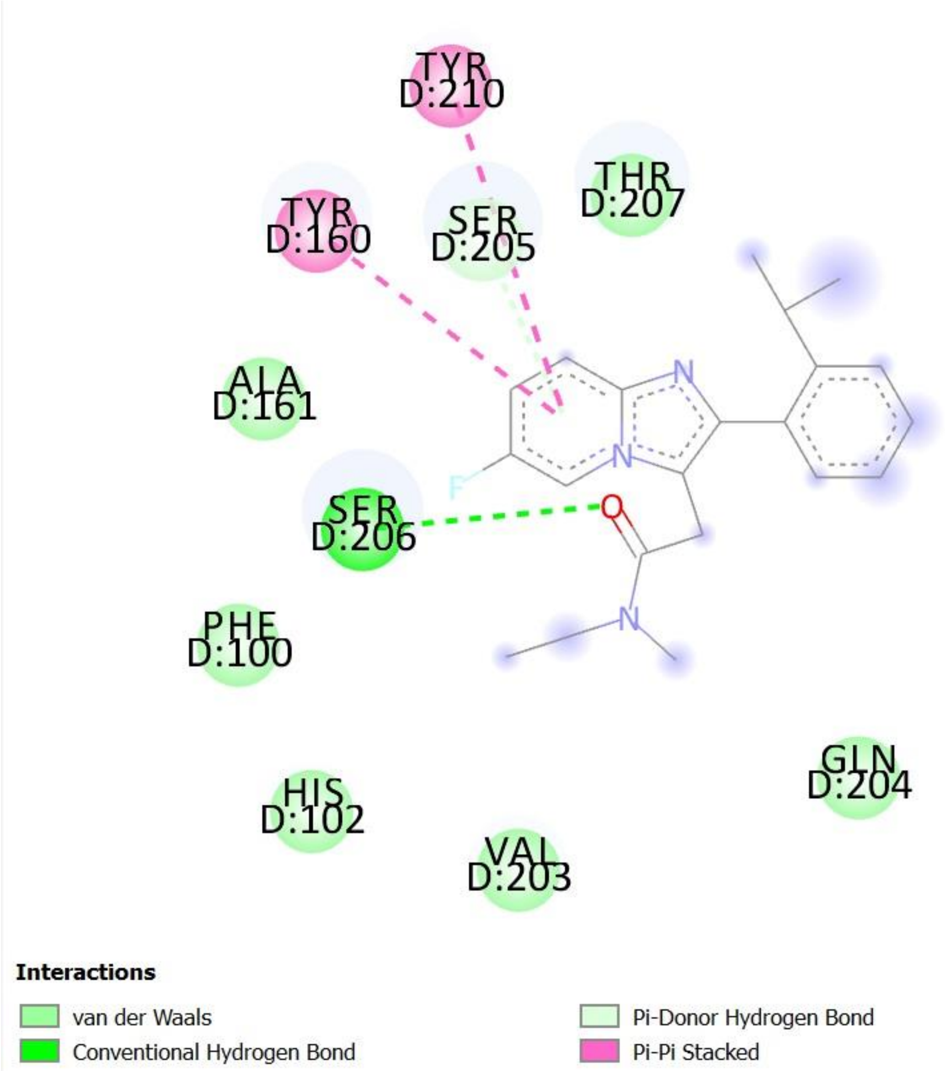

Figure S9. Two-dimensional representation of the interaction between molecule A7 and amino acids inside ionotropic $GABA_A$ receptor.

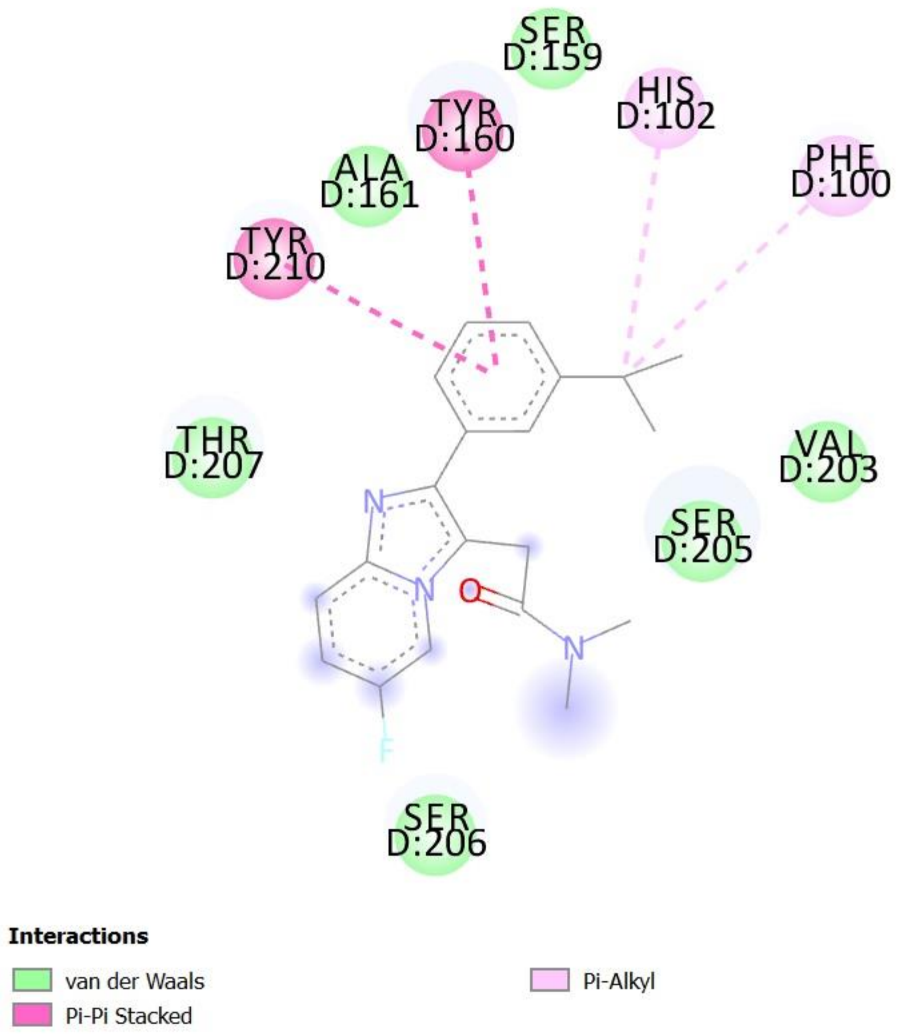

Figure S10. Two-dimensional representation of the interaction between molecule A8 and amino acids inside ionotropic $GABA_A$ receptor.

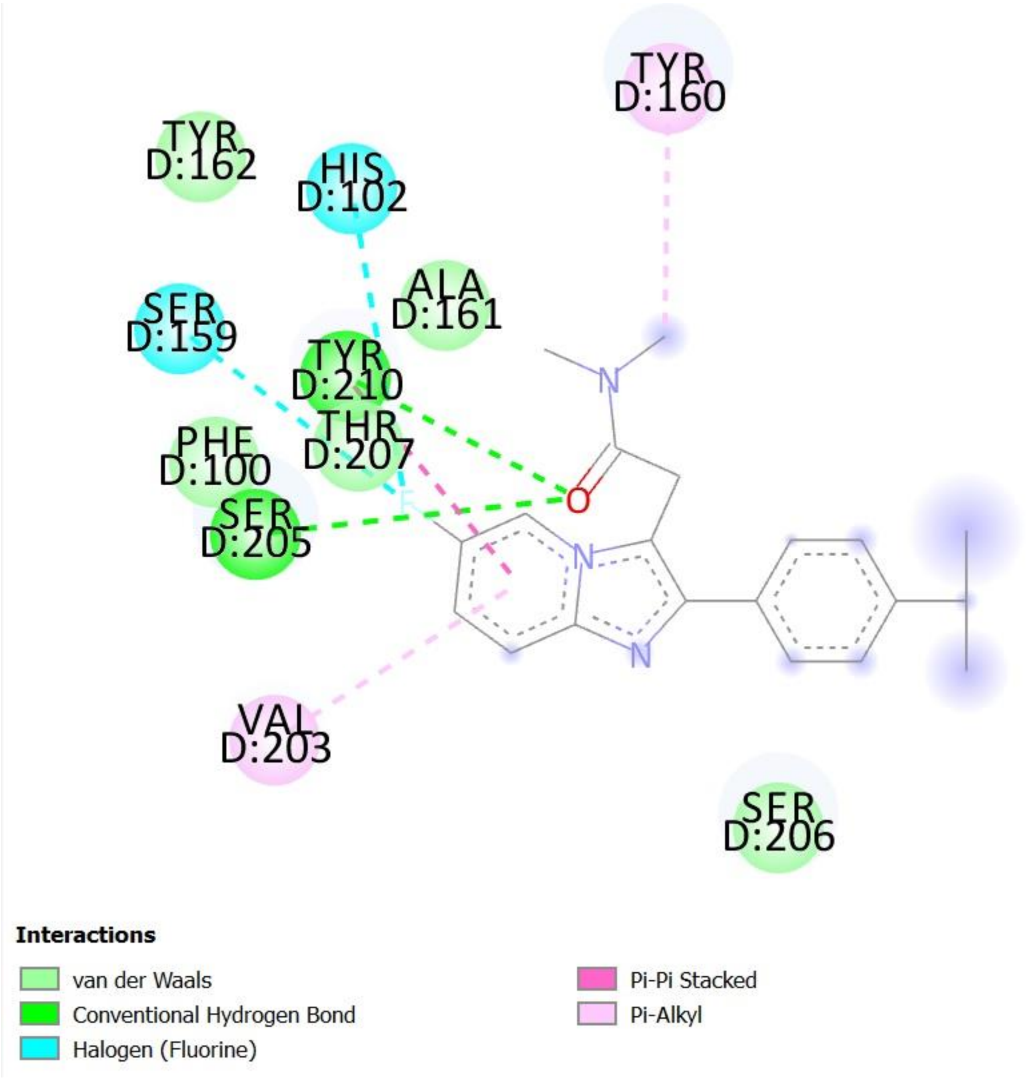

Figure S11. Two-dimensional representation of the interaction between molecule A9 and amino acids inside ionotropic $GABA_A$ receptor.

Table S5. Calculated physical-chemical parameters for drug-likeness evaluation.

Molecule	A	A1	A2	A3	A4	A5	A5	A7	A8	A9
MW	297.33	311.35	311.35	311.35	325.38	325.38	325.38	353.43	339.41	339.41
#Heavy atoms	22	23	23	23	24	24	24	26	25	25
#Aromatic heavy atoms	15	15	15	15	15	15	15	15	15	15
Fraction Csp3	0.18	0.22	0.22	0.22	0.26	0.26	0.26	0.33	0.3	0.3
#Rotatabl e bonds	4	4	4	4	5	5	5	6	5	5
#H-bond acceptors	3	3	3	3	3	3	3	3	3	3
#H-bond donors	0	0	0	0	0	0	0	0	0	0
MR	83.07	88.03	88.03	88.03	92.84	92.84	92.84	102.46	97.65	97.65
TPSA	37.61	37.61	37.61	37.61	37.61	37.61	37.61	37.61	37.61	37.61
iLOGP	2.78	3.07	2.98	3.05	2.91	3.33	3.57	3.2	3.55	3.4
XLOGP3	2.96	3.32	3.32	3.32	3.75	3.75	3.75	4.45	4.08	4.08
WLOGP	3.19	3.5	3.5	3.5	3.75	3.75	3.75	4.7	4.31	4.31
MLOGP	2.27	2.51	2.51	2.51	2.74	2.74	2.74	3.18	2.96	2.96
Silicos-IT Log P	2.74	3.25	3.25	3.25	3.64	3.64	3.64	4.26	3.86	3.86
Consensu s Log P	2.79	3.13	3.11	3.13	3.36	3.44	3.49	3.96	3.75	3.72
ESOL Log S	-3.79	-4.08	-4.08	-4.08	-4.35	-4.35	-4.35	-4.87	-4.63	-4.63
ESOL Solubility (mg/ml)	4.84E-02	2.59E-02	2.59E-02	2.59E-02	1.45E-02	1.45E-02	1.45E-02	4.82E-03	7.98E-03	7.98E-03
ESOL Solubility (mol/l)	1.63E-04	8.31E-05	8.31E-05	8.31E-05	4.44E-05	4.44E-05	4.44E-05	1.36E-05	2.35E-05	2.35E-05
ESOL Class	Solubl e	Moder ately solubl e	Moder ately solubl e	Moder ately solubl e	Moder ately solubl e	Moder ately solubl e	Moder ately solubl e	Moder ately solubl e	Moder ately solubl e	Moder ately solubl e
Ali Log S	-3.41	-3.79	-3.79	-3.79	-4.23	-4.23	-4.23	-4.96	-4.57	-4.57
Ali Solubility (mg/ml)	1.15E-01	5.09E-02	5.09E-02	5.09E-02	1.91E-02	1.91E-02	1.91E-02	3.89E-03	9.03E-03	9.03E-03

Ali Solubility (mol/l)	3.87E-04	1.64E-04	1.64E-04	1.64E-04	5.86E-05	5.86E-05	5.86E-05	1.10E-05	2.66E-05	2.66E-05
Ali Class	Soluble	Soluble	Soluble	Soluble	Moderately soluble	Moderately soluble	Moderately soluble	Moderately soluble	Moderately soluble	Moderately soluble
Silicos-IT LogSw	-5.34	-5.72	-5.72	-5.72	-6.11	-6.11	-6.11	-6.53	-6.14	-6.14
Silicos-IT Solubility (mg/ml)	1.37E-03	5.97E-04	5.97E-04	5.97E-04	2.50E-04	2.50E-04	2.50E-04	1.04E-04	2.48E-04	2.48E-04
Silicos-IT Solubility (mol/l)	4.60E-06	1.92E-06	1.92E-06	1.92E-06	7.69E-07	7.69E-07	7.69E-07	2.94E-07	7.29E-07	7.29E-07
Silicos-IT class	Moderately soluble	Moderately soluble	Moderately soluble	Moderately soluble	Poorly soluble	Poorly soluble	Poorly soluble	Poorly soluble	Poorly soluble	Poorly soluble
GI absorption	High	High	High	High	High	High	High	High	High	High
BBB permeant	Yes	Yes	Yes	Yes	Yes	Yes	Yes	Yes	Yes	Yes
Pgp substrate	No	No	No	No	No	No	No	No	No	No
CYP1A2 inhibitor	Yes	Yes	Yes	Yes	Yes	Yes	Yes	Yes	Yes	Yes
CYP2C19 inhibitor	Yes	Yes	Yes	Yes	Yes	Yes	Yes	Yes	Yes	Yes
CYP2C9 inhibitor	Yes	Yes	Yes	Yes	Yes	Yes	Yes	Yes	Yes	Yes
CYP2D6 inhibitor	Yes	Yes	Yes	Yes	Yes	Yes	Yes	Yes	Yes	Yes
CYP3A4 inhibitor	No	Yes	Yes	Yes	Yes	Yes	Yes	Yes	Yes	Yes
log Kp (cm/s)	-6.01	-5.84	-5.84	-5.84	-5.62	-5.62	-5.62	-5.3	-5.47	-5.47
Lipinski #violations	0	0	0	0	0	0	0	0	0	0
Ghose #violations	0	0	0	0	0	0	0	0	0	0
Veber #violations	0	0	0	0	0	0	0	0	0	0

Egan #violations	0	0	0	0	0	0	0	0	0	0
Muegge #violations	0	0	0	0	0	0	0	0	0	0
Bioavailability Score	0.55	0.55	0.55	0.55	0.55	0.55	0.55	0.55	0.55	0.55
PAINS #alerts	0	0	0	0	0	0	0	0	0	0
Brenk #alerts	0	0	0	0	0	0	0	0	0	0
Leadlikeness #violations	0	0	0	0	1	1	1	2	1	1
Synthetic Accessibility	2.76	2.9	2.93	2.88	3.02	3.02	2.97	3.22	3.13	3.08

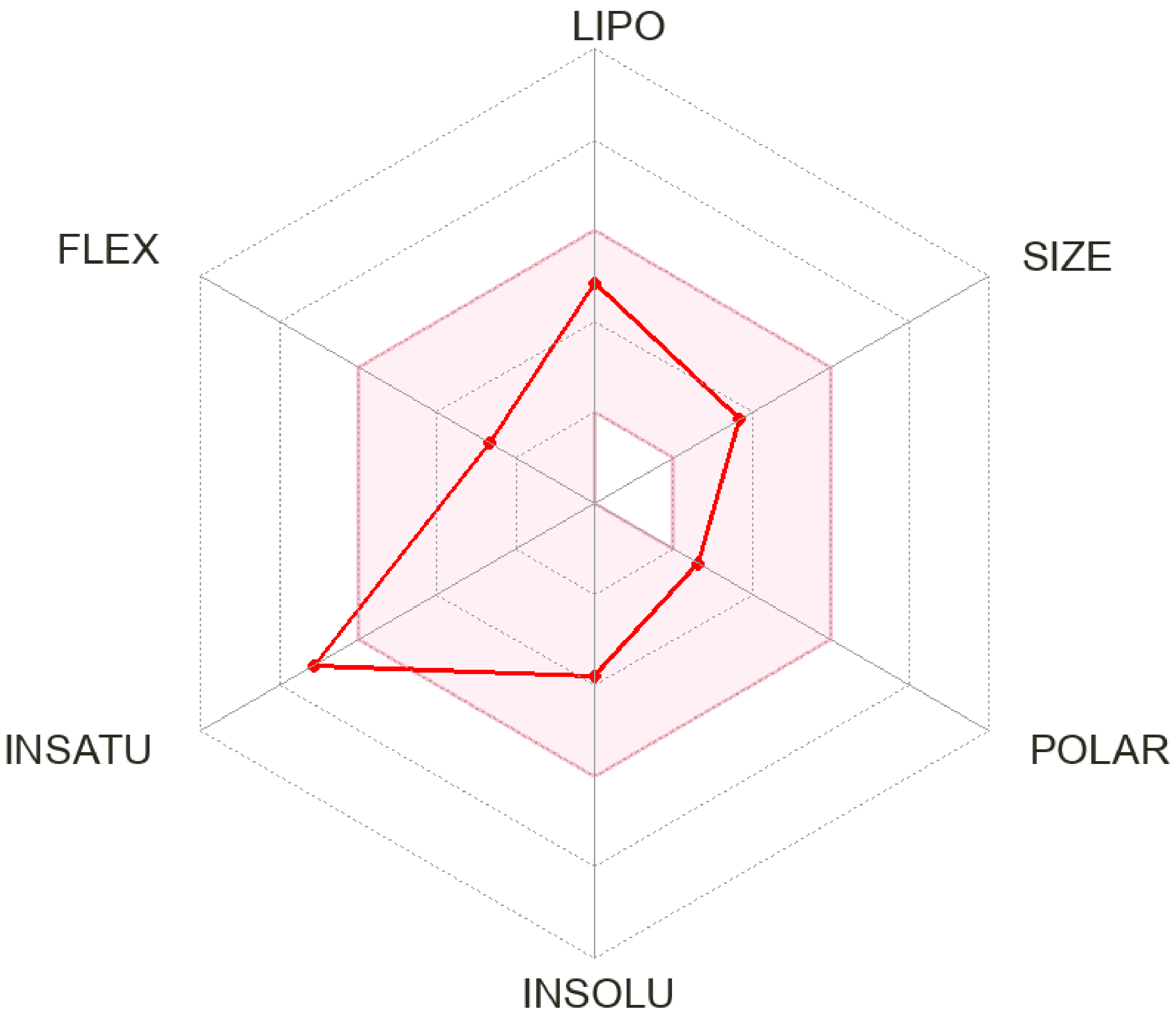

Figure S12. Graphical representations of molecule A important physico-chemical features – Bioavailability Radar. The pink area represents the optimal range for each properties (lipophilicity: XLOGP3 between −0.7 and +5.0, size: MW between 150 and 500 g/mol, polarity: TPSA between 20 and 130 Å2, solubility: log S not higher than 6, saturation:

fraction of carbons in the sp3 hybridization not less than 0.25, and flexibility: no more than 9 rotatable bonds.

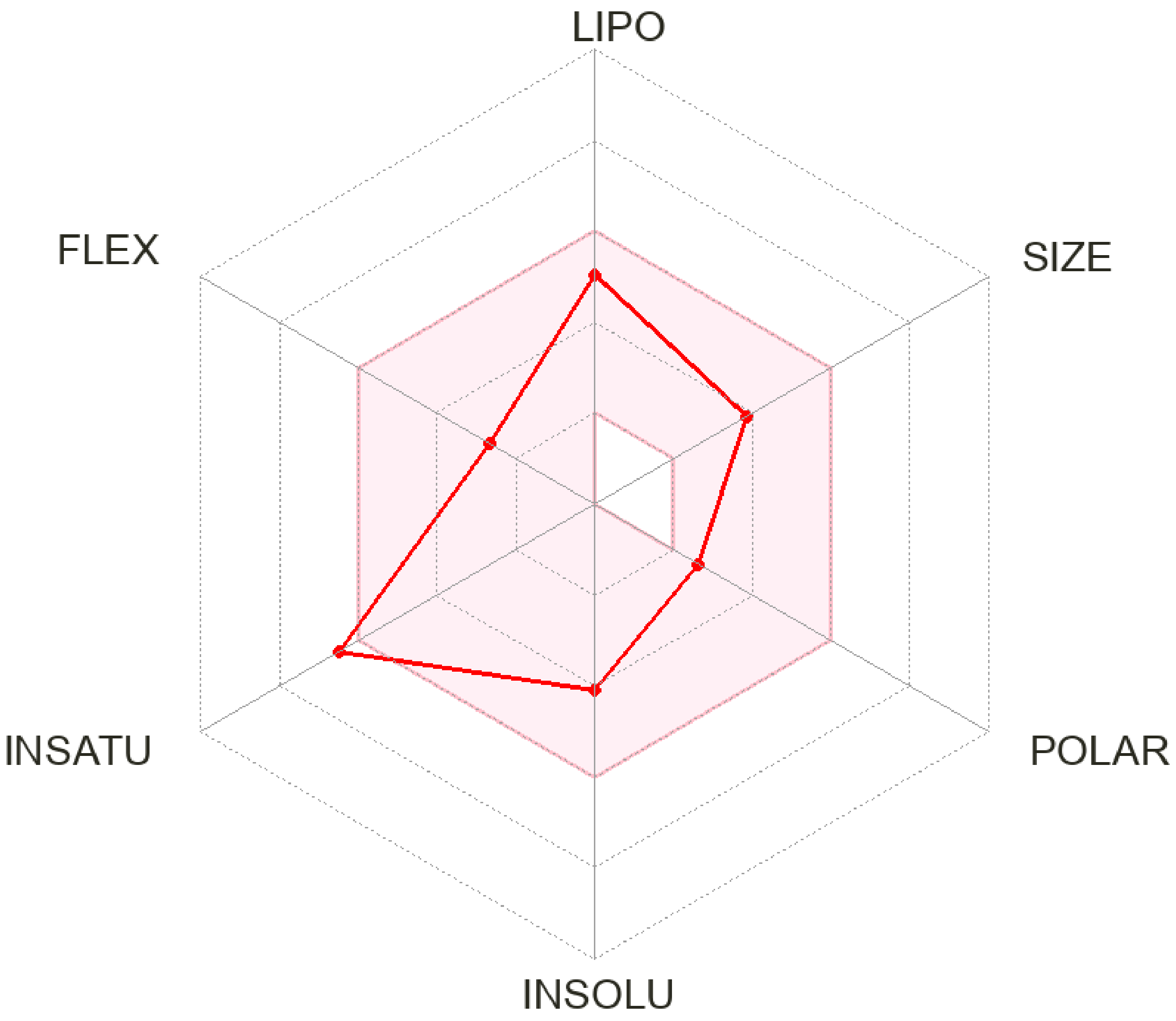

Figure S13. Graphical representations of molecule A1 important physico-chemical features – Bioavailability Radar. The pink area represents the optimal range for each

properties (lipophilicity: XLOGP3 between −0.7 and +5.0, size: MW between 150 and 500 g/mol, polarity: TPSA between 20 and 130 Å2, solubility: log S not higher than 6, saturation: fraction of carbons in the sp3 hybridization not less than 0.25, and flexibility: no more than 9 rotatable bonds.

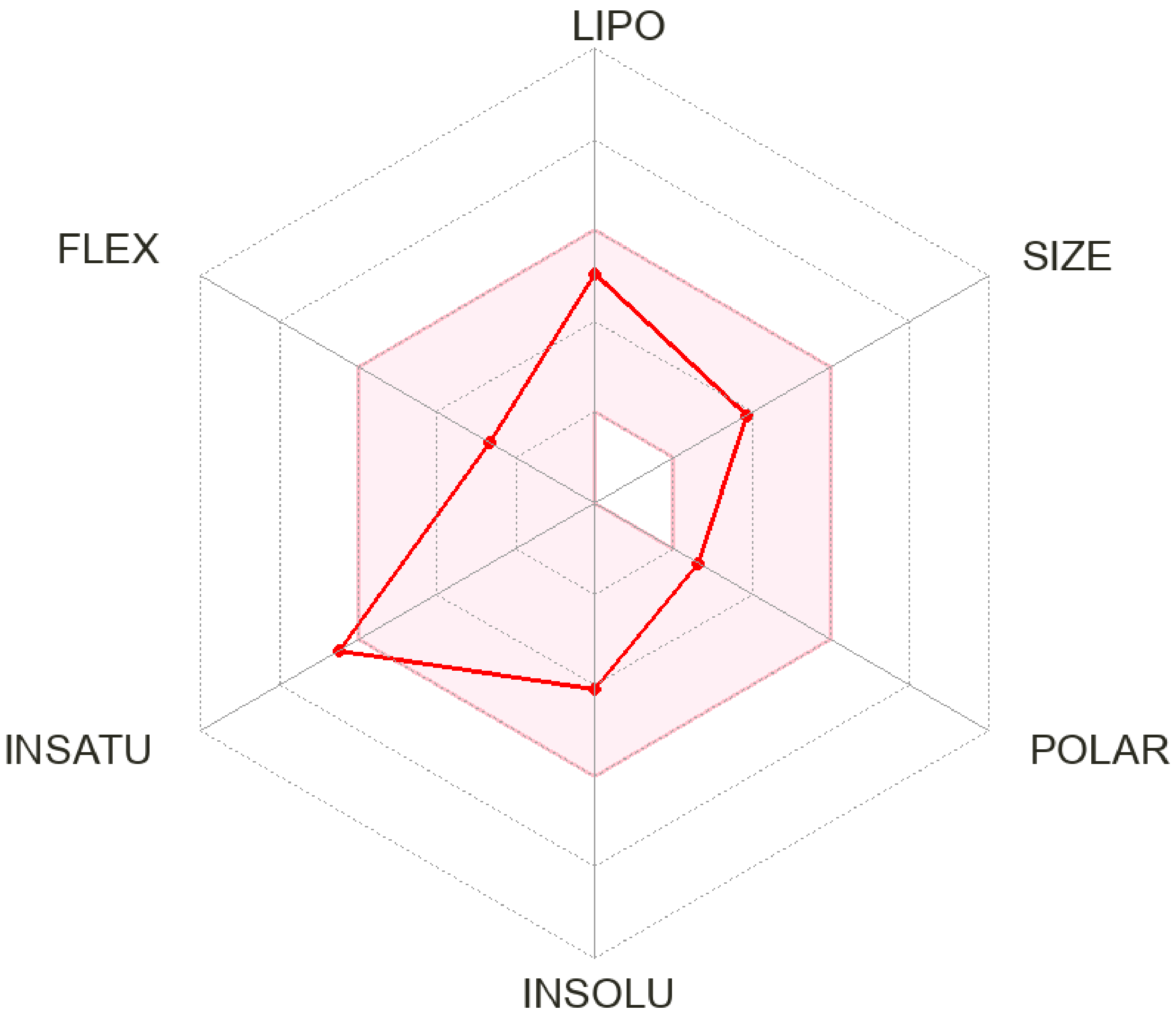

Figure S14. Graphical representations of molecule A2 important physico-chemical features – Bioavailability Radar. The pink area represents the optimal range for each properties (lipophilicity: XLOGP3 between −0.7 and +5.0, size: MW between 150 and 500 g/mol, polarity: TPSA between 20 and 130 Å2, solubility: log S not higher than 6, saturation: fraction of carbons in the sp3 hybridization not less than 0.25, and flexibility: no more than 9 rotatable bonds.

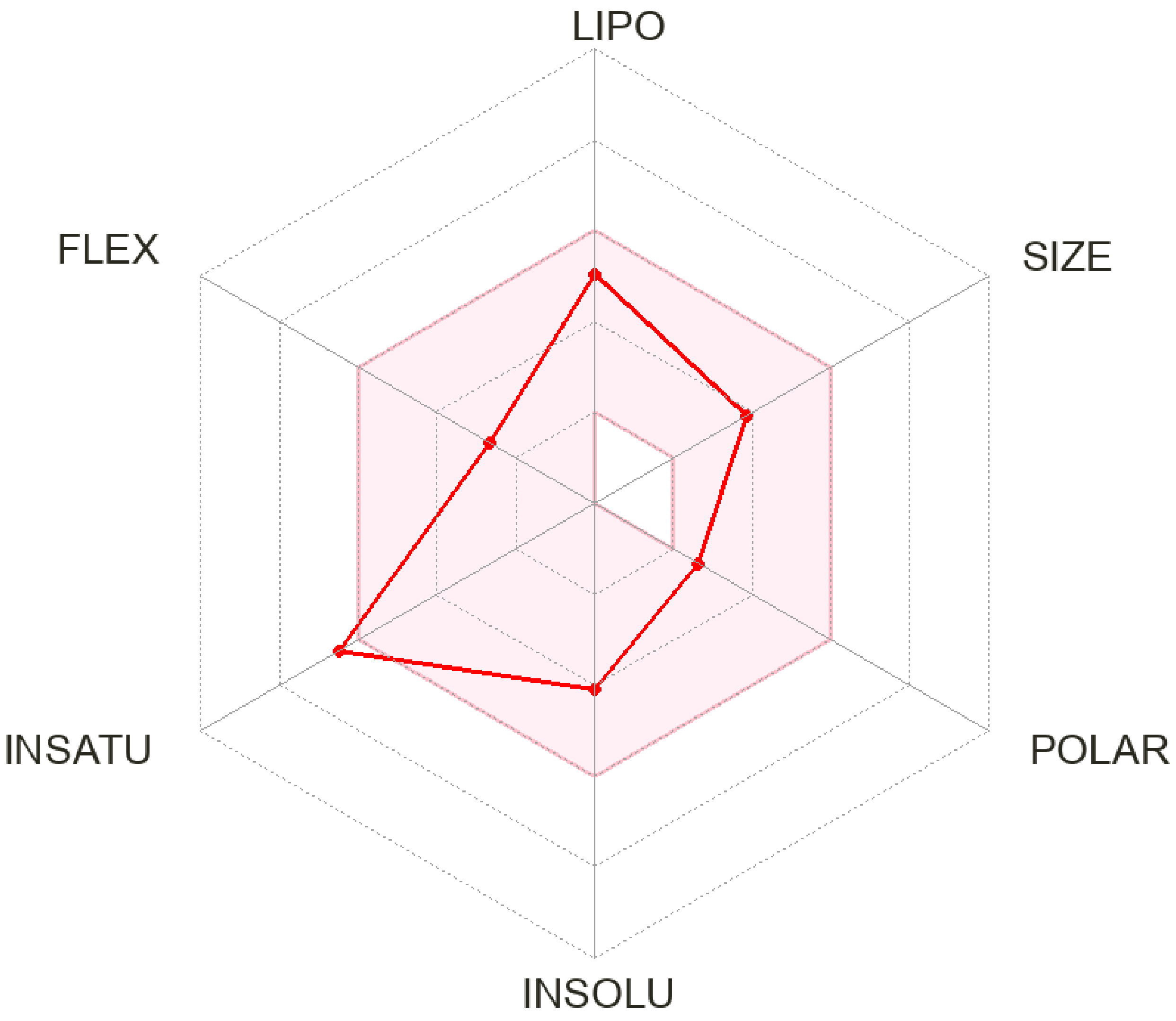

Figure S15. Graphical representations of molecule A3 important physico-chemical features – Bioavailability Radar. The pink area represents the optimal range for each properties (lipophilicity: XLOGP3 between −0.7 and +5.0, size: MW between 150 and 500 g/mol, polarity: TPSA between 20 and 130 Å2, solubility: log S not higher than 6,

saturation: fraction of carbons in the sp3 hybridization not less than 0.25, and flexibility: no more than 9 rotatable bonds.

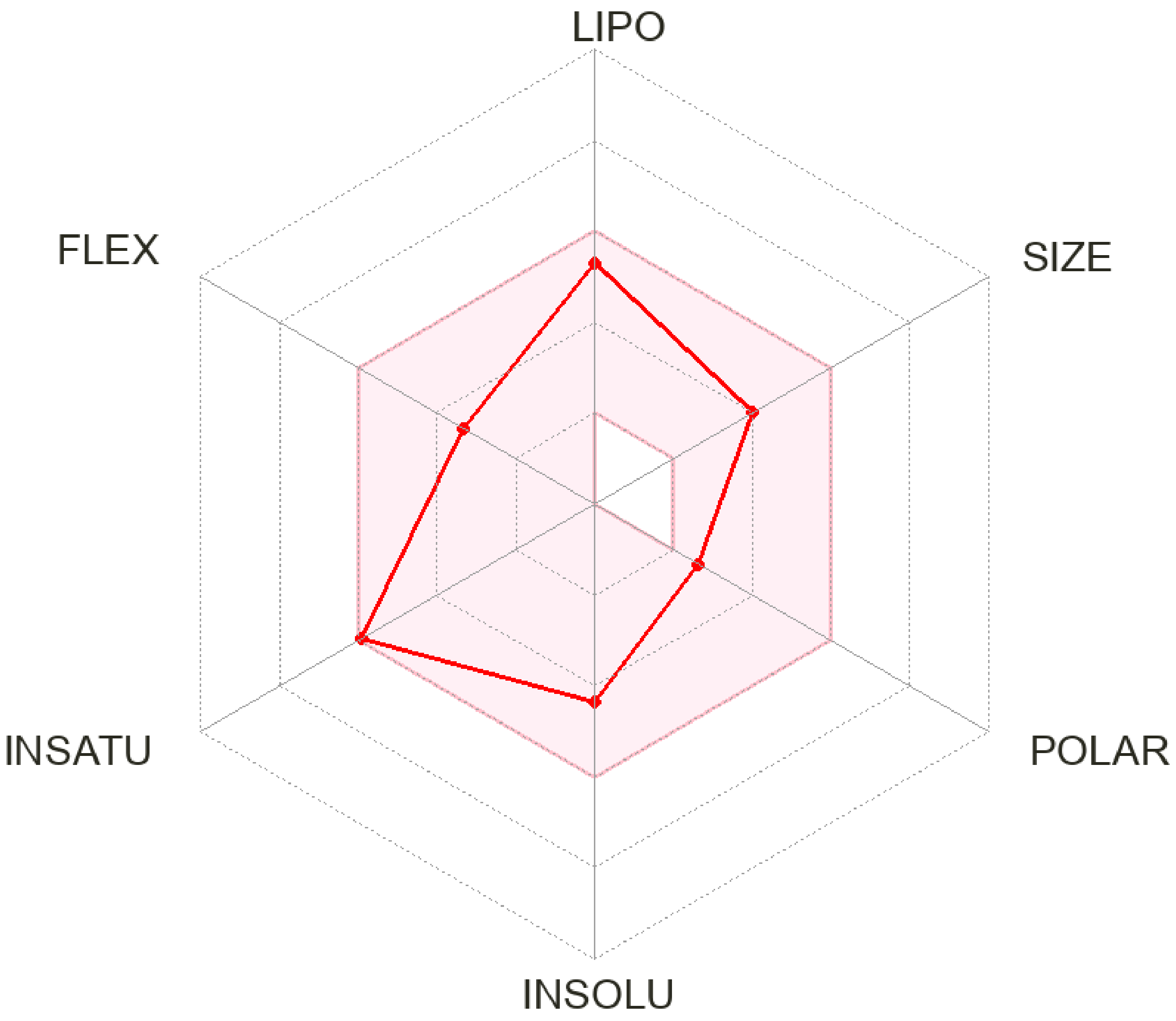

Figure S16. Graphical representations of molecule A4 important physico-chemical features – Bioavailability Radar. The pink area represents the optimal range for each

properties (lipophilicity: XLOGP3 between −0.7 and +5.0, size: MW between 150 and 500 g/mol, polarity: TPSA between 20 and 130 Å2, solubility: log S not higher than 6, saturation: fraction of carbons in the sp3 hybridization not less than 0.25, and flexibility: no more than 9 rotatable bonds.

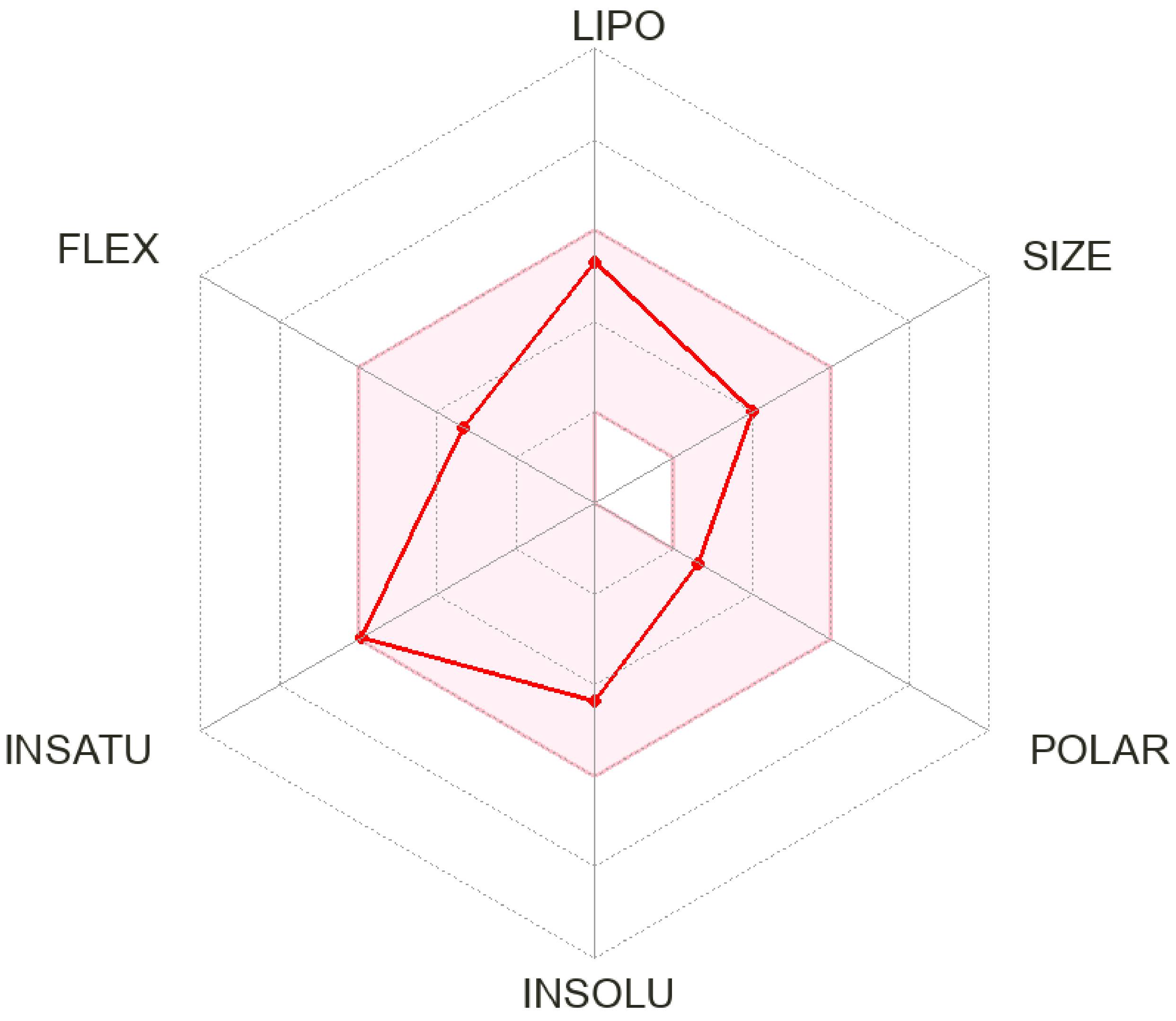

Figure S17. Graphical representations of molecule A5 important physico-chemical features – Bioavailability Radar. The pink area represents the optimal range for each properties (lipophilicity: XLOGP3 between −0.7 and +5.0, size: MW between 150 and 500 g/mol, polarity: TPSA between 20 and 130 Å2, solubility: log S not higher than 6, saturation: fraction of carbons in the sp3 hybridization not less than 0.25, and flexibility: no more than 9 rotatable bonds.

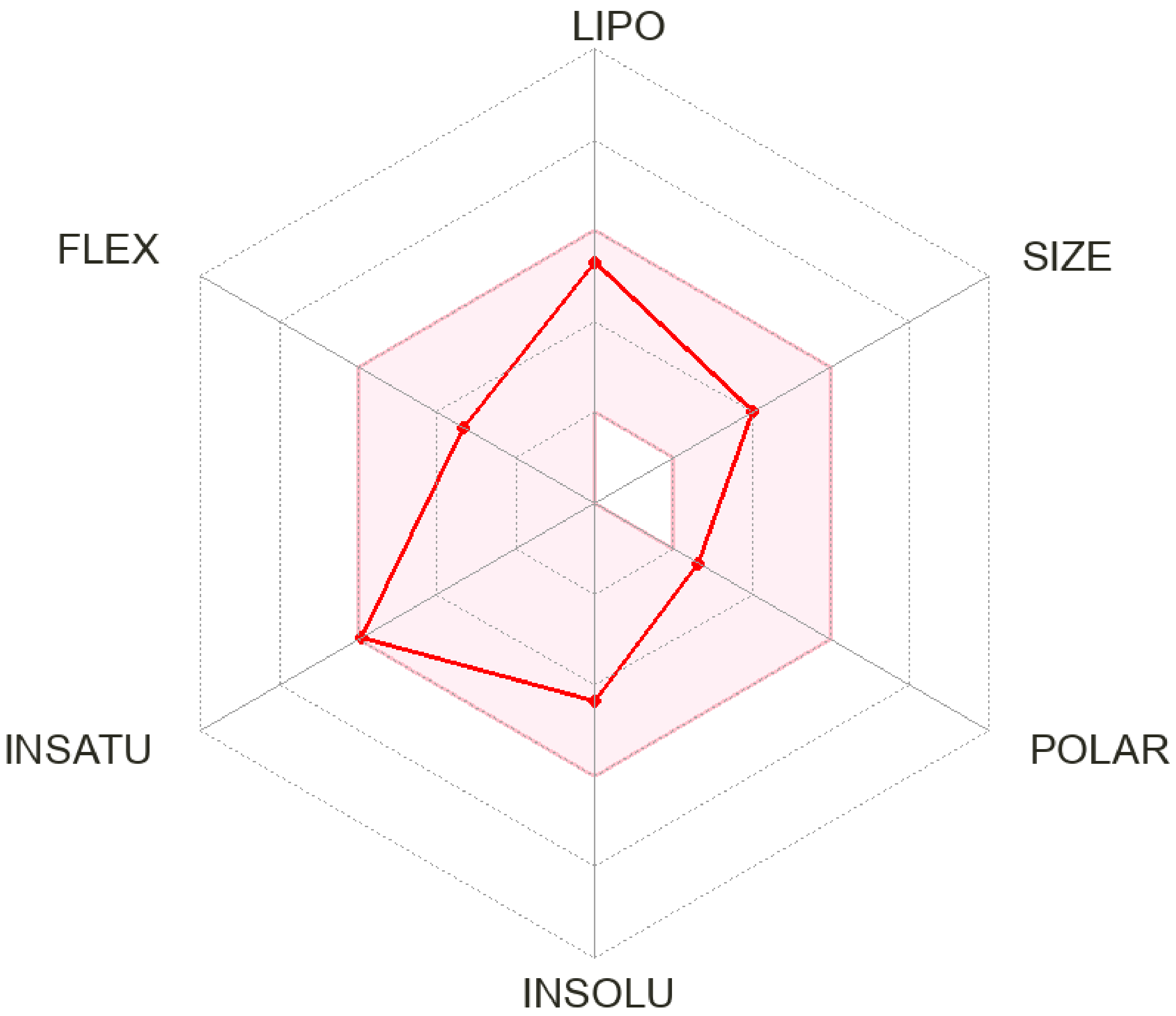

Figure S18. Graphical representations of molecule A6 important physico-chemical features – Bioavailability Radar. The pink area represents the optimal range for each properties (lipophilicity: XLOGP3 between −0.7 and +5.0, size: MW between 150 and 500 g/mol, polarity: TPSA between 20 and 130 Å2, solubility: log S not higher than 6,

saturation: fraction of carbons in the sp3 hybridization not less than 0.25, and flexibility: no more than 9 rotatable bonds.

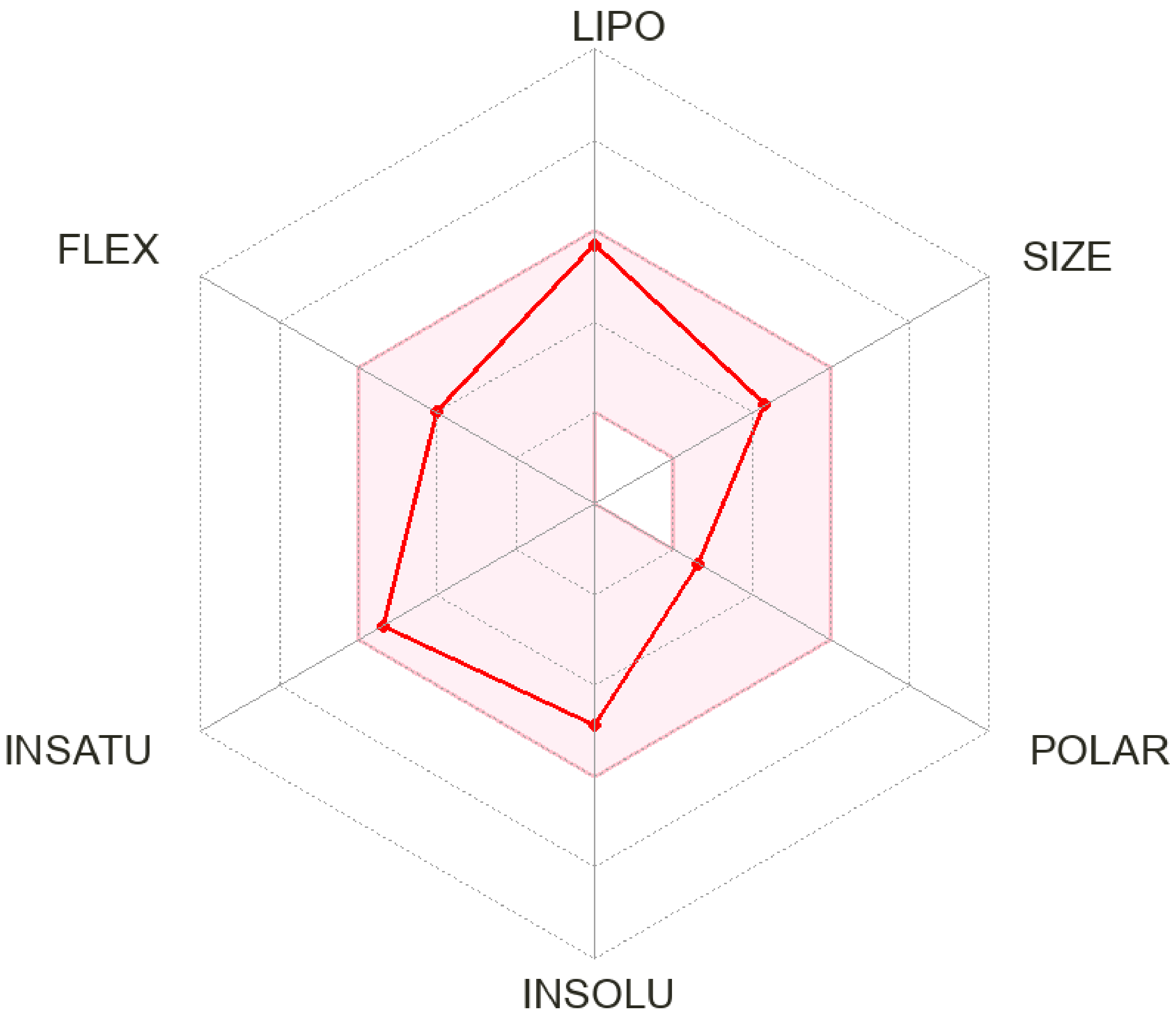

Figure S19. Graphical representations of molecule A7 important physico-chemical features – Bioavailability Radar. The pink area represents the optimal range for each

properties (lipophilicity: XLOGP3 between −0.7 and +5.0, size: MW between 150 and 500 g/mol, polarity: TPSA between 20 and 130 Å2, solubility: log S not higher than 6, saturation: fraction of carbons in the sp3 hybridization not less than 0.25, and flexibility: no more than 9 rotatable bonds.

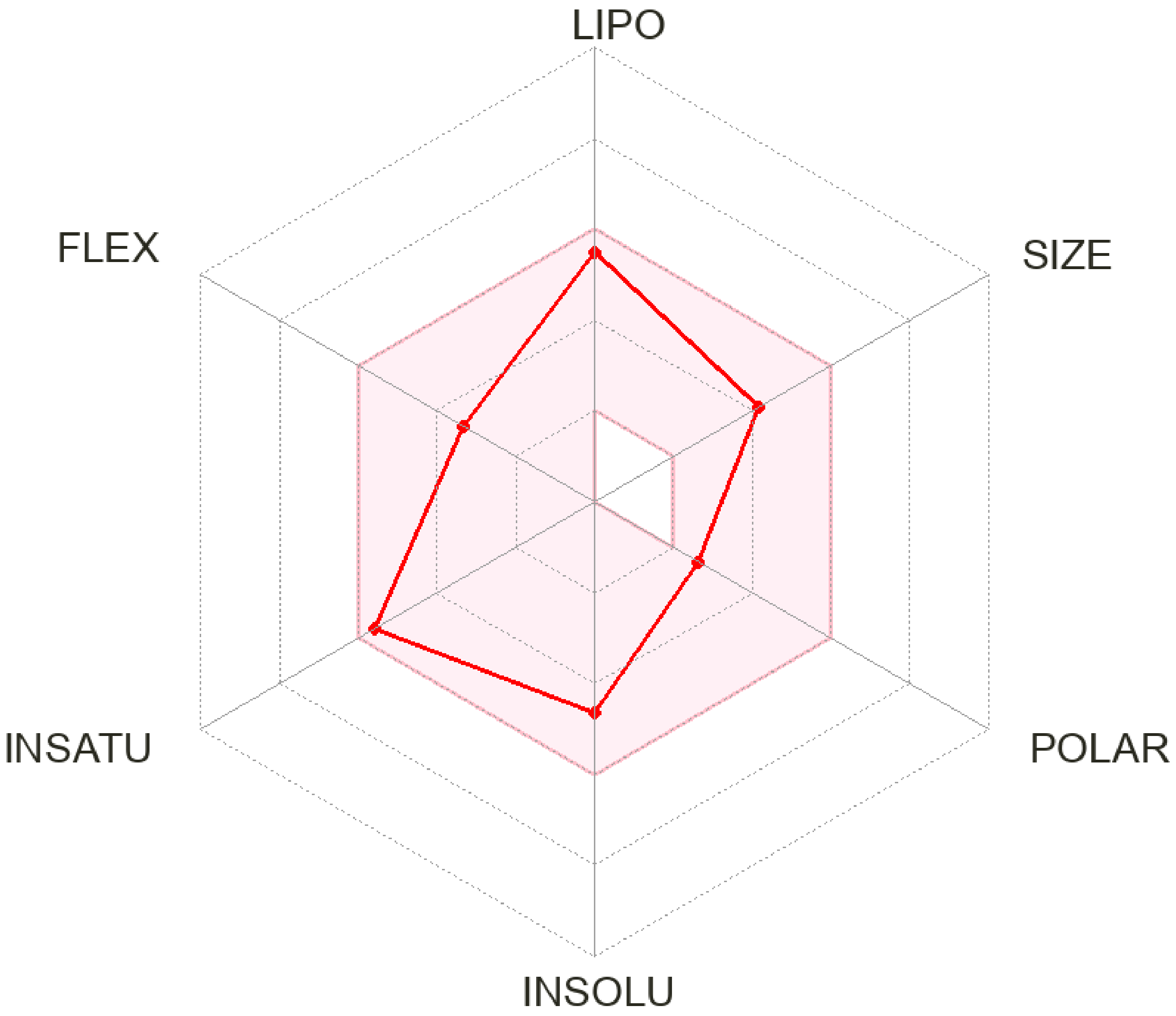

Figure S20. Graphical representations of molecule A8 important physico-chemical features – Bioavailability Radar. The pink area represents the optimal range for each properties (lipophilicity: XLOGP3 between −0.7 and +5.0, size: MW between 150 and 500 g/mol, polarity: TPSA between 20 and 130 Å2, solubility: log S not higher than 6, saturation: fraction of carbons in the sp3 hybridization not less than 0.25, and flexibility: no more than 9 rotatable bonds.

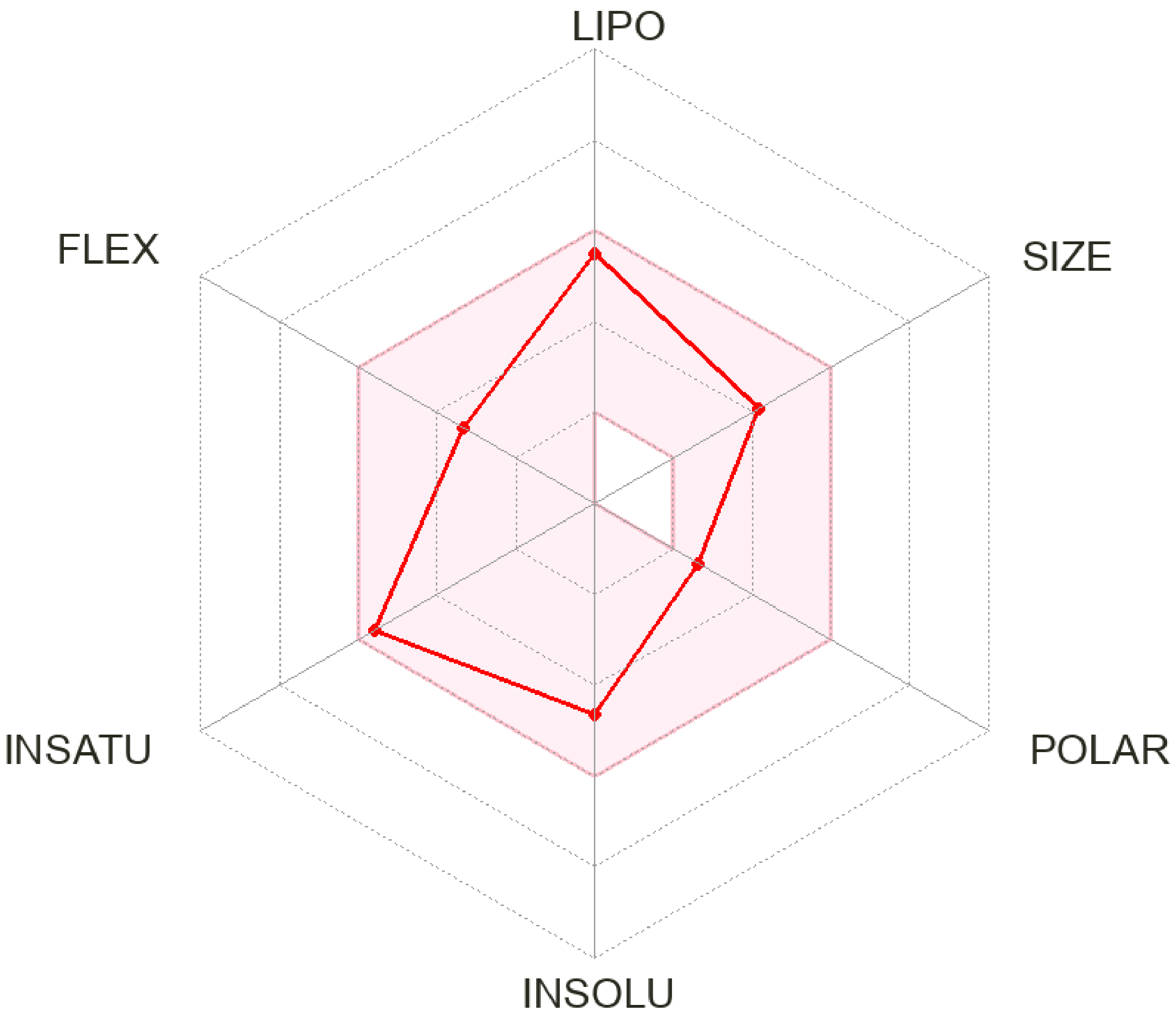

Figure S21. Graphical representations of molecule A9 important physico-chemical features – Bioavailability Radar. The pink area represents the optimal range for each properties (lipophilicity: XLOGP3 between −0.7 and +5.0, size: MW between 150 and 500 g/mol, polarity: TPSA between 20 and 130 Å2, solubility: log S not higher than 6,

saturation: fraction of carbons in the sp3 hybridization not less than 0.25, and flexibility: no more than 9 rotatable bonds.
